# Non-equilibrium correlated electron dynamics in triangular molecular assemblies

**DOI:** 10.1038/s41467-026-75051-3

**Published:** 2026-07-08

**Authors:** Chao Li, Vladislav Pokorný, Prokop Hapala, Martin Žonda, Thilo Glatzel, Ping Zhou, Silvio Decurtins, Shi-Xia Liu, Fengqi Song, Ernst Meyer, Rémy Pawlak

**Affiliations:** 1 Institute of Atom Manufacturing, Nanjing University, Nanjing, China; 2 Department of Physics, WSS Research Centre for Molecular Quantum Systems, University of Basel, Basel, Switzerland; 3 Institute of Physics (FZU), Czech Academy of Sciences, Prague, Czech Republic; 4 Department of Condensed Matter Physics, Faculty of Mathematics and Physics, Charles University, Prague, Czech Republic; 5 Department of Chemistry, Biochemistry and Pharmaceutical Sciences, W. Inäbnit Laboratory for Molecular Quantum Materials and WSS Research Centre for Molecular Quantum Systems, University of Bern, Bern, Switzerland

**Keywords:** Molecular electronics, Superconducting devices

## Abstract

Understanding and controlling charge states at the level of individual electrons in molecular assemblies is often hindered by system complexity and the lack of reliable theoretical framework to accurately model the underlying many-body non-equilibrium electron dynamics. To address this challenge, here we construct well-defined molecular trimers and hexamers of tetrabromo-tetraazapyrene molecules by scanning probe manipulation and show that the electron dynamics can be completely rationalized using the Anderson impurity model and master equation approach. Our analysis demonstrates that such a treatment, which goes beyond the standard single-particle picture, is essential for understanding this class of molecular systems. The model not only quantitatively reproduces all features visible in measured differential conductance maps, but also uncovers the mechanism for negative differential conductance, which arises from the non-equilibrium occupancy of collective charge states. The analysis also reveals that the charging rings are not necessarily associated with changes in the total charge of the cluster, but often originate from internal charge rearrangements between the sites. These findings provide insight into fundamental quantum many-body effects in strongly interacting molecular systems and open pathways for engineering electronic phases by controlling cluster topology and the electrostatic environment.

## Abstract (web summary)

Molecular systems can exhibit Coulomb rings in scanning tunneling microscopy images. Here, the authors construct molecular trimers and hexamers and use a many-body impurity Anderson model and master equation solver to simulate the Coulomb ring patterns and underlying non-equilibrium charge dynamics.

## Introduction

The relentless drive to miniaturize electronic components has brought us to scales where quantum phenomena and atomic-level details dominate electron dynamics. Under such conditions, strong Coulomb interactions, combined with spatial confinement, yield emergent behaviors markedly different from those of bulk materials. Molecular assemblies, due to their atomic precision and sub-nanometer size, provide an ideal platform to explore the complex interplay of phenomena arising from electron-electron interactions, such as the Kondo effect, Coulomb blockade, and inter-molecular capacitive couplings^1–4^. Unlike semiconductor junctions and quantum dots, these assemblies can also be mass-produced with atomic precision via environmentally friendly and energy-efficient methods of organic synthesis^5,6^.

Previous investigations of charging effects and tunneling (d*I*/d*V*) spectra in molecular systems have often been limited to a qualitative understanding due to the large complexity of extended systems like 2D layers. Interpretations have typically relied on a simplistic single-particle picture, where the electric field of the scanning probe tip gates a single molecular orbital across the Fermi level^7,8^. Consequently, the characteristic Coulomb rings observed in d*I*/d*V* maps have been universally interpreted as signatures of a change in the system’s total charge. This picture, however, fails to capture the collective electronic behavior inherent to strongly correlated quantum systems. This leaves a critical gap in our ability to model and engineer charge states at the nanoscale, hindering the development of molecular assemblies as compelling platforms for computing paradigms beyond conventional electronics, such as the concept of molecular quantum cellular automata, where information is processed through local capacitive couplings between precisely arranged molecules^9–11^.

Here, we bridge the gap by combining atomically precise molecular manipulation with a rigorous theoretical framework, allowing us to fully understand and quantitatively simulate the experimental results. We first construct a *C*_3_-symmetric trimer of radical 4,5,9,10-tetrabromo-1,3,6,8-tetraazapyrene (TBTAP) molecules on a superconducting Pb(111) substrate, manipulated with atomic precision to form a compact molecular cluster. In our previous works, we demonstrated that TBTAP molecules exist as radicals when adsorbed on both Ag(111) and Pb(111) surfaces^12,13^, making them an ideal platform for investigating electron-electron interactions within designed structures^14^. The trimer is intentionally designed to capture nontrivial charging behavior while being simple enough for an exact many-body treatment, which is impossible for larger assemblies due to the exponential growth of the Hilbert space. Triangular trimer structures were also studied previously in different contexts and they exhibit a variety of interesting transport phenomena^15–21^. Using low-temperature scanning tunneling spectroscopy, we directly visualize discharging processes and characteristic Coulomb ring patterns associated with single-electron charging events, including the onset of robust negative differential conductance (NDC) near the center of the trimer. NDC was previously observed only in extended systems like two-dimensional heterostructures^22–25^ and layers^7,26–28^ or systems with limited control over the geometry, such as break and tunnel junctions^29–34^. The theoretical simulations of the charge dynamics using the many-body impurity Anderson model and master equation solver achieve perfect agreement with experimental observations, enabling us to move beyond the simplistic single-particle view and allowing us to present the fully quantitative description of d*I*/d*V* spectra of a molecular cluster.

In this work, we show that the charging behavior is independent of the underlying superconductivity of the substrate and leads to two fundamental discoveries. First, we find that a significant number of the (dis)charging rings and conductance discontinuities do not correspond to a change in the total charge of the trimer (although some do). Instead, they originate from subtle intra-cluster charge rearrangements, where electrons are redistributed among the molecules without altering the net charge. This finding challenges the prevailing interpretation of charging phenomena in molecular systems. Second, the simulation reveals that a proper explanation of the transport spectra requires considering the collective many-body states of the entire cluster and, crucially, their non-equilibrium populations. It is precisely this non-equilibrium occupancy, stabilized by the capacitive coupling, that gives rise to unexpected features, including NDC. Moreover, by expanding the molecular cluster to a triangular hexamer assembly, we highlight how cluster topology can be used to engineer electronic responses. Our work thus provides a complete, atomically-resolved picture of the non-equilibrium many-body dynamics that govern quantum transport at the ultimate limit of miniaturization.

## Results and discussion

### Triangular trimer assemblies and their discharging behavior

**Fig. 1:**
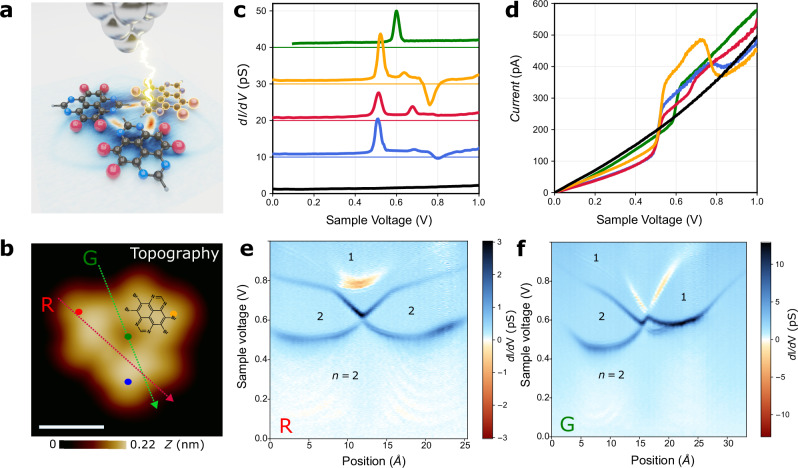
Triangular trimer structures of TBTAP molecules. **a** Schematic diagram showing the design of the molecular trimer structure. The local electric field of an STM tip can discharge anionic TBTAP molecules (blue) to neutral (orange), leading to observation of Coulomb rings in d*I*/d*V* maps. The molecules are coupled via inter-site Coulomb interactions. **b** High-resolution STM image (*I *= 100 pA, *V*_s _= 600 mV) of a triangular structure constructed from three TBTAP molecules. The scale bar is 1 nm. Tunneling spectra (**c**) and current (**d**) measured at positions given by color points in (**b**). The black line represents the spectrum and current measured over a pure Pb surface. Spectra are vertically shifted for clarity. **e** Position-dependent d*I*/d*V* spectra along the red line shown in (**b**), which passes over the centers of two molecules. The total number of electrons *n* decreases from two to one with increasing sample voltage, as inferred from the simulations. Note that not all peaks in d*I*/d*V* correspond to a change of the total charge on the trimer. **f** Position-dependent d*I*/d*V* spectra along the green line shown in (**b**), which connects the center of the trimer and a center of a molecule.

We achieved a precise assembly of triangular molecular trimers by manipulating TBTAP molecules on the Pb(111) surface using lateral manipulation (Supplementary Fig. 2). The molecules form an equilateral triangle with a 1 nm distance between the centers of the molecules (Fig. 1a). The trimer is stabilized through a combination of hydrogen bonding and N⋯Br halogen bonding, in agreement with a DFT simulation (Supplementary Fig. 4), creating a chiral structure. The chirality in this case is the result of the incommensurate size of the TBTAP molecules with respect to the surface lattice constant of Pb(111). Low-energy spectra were measured at temperature ${T}_{\exp }=2.6$ K and show two pairs of well-developed Yu-Shiba-Rusinov (YSR) states inside the superconducting gap at *V*_s_ = ± 1.85 mV and ±1.95 mV (Supplementary Fig. 11). The observation of YSR states proves the presence of additional charge on the molecular cluster.

Tunneling (d*I*/d*V*) spectra and current were also recorded at higher voltages at positions marked by colored dots in Fig. 1b. As shown in Fig. 1c, the d*I*/d*V* spectra exhibit sharp peaks at *V*_s_ = 520 mV on individual molecules (yellow, red and blue lines) and at *V*_s_ = 590 mV at the center of the trimer (green line). These peaks, that we attribute to changes of the charge state of the trimer, gradually shift toward the Fermi energy as the tip height decreases (see Supplementary Fig. 5), consistent with the tip-induced electric field effect^12,35,36^. In addition to these sharp peaks, NDC regions, characterized by dips in the spectra, were observed at energies close to *V*_s_ = 760 mV. These regions are marked by drops in current with increasing voltage, Fig. 1d. Figure 1e, f display position-dependent d*I*/d*V* spectra along the red and green lines indicated in Fig. 1b, respectively. Data show bands of high positive and high negative conductance, which correspond to jumps in the tip current, which are attributed to a gain or loss of an electron at the given molecule.

**Fig. 2:**
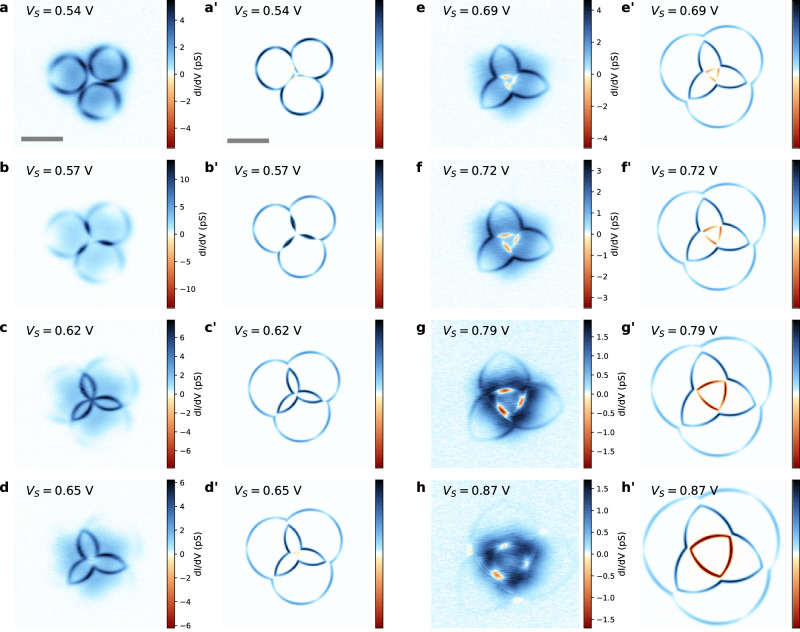
Discharging rings in a molecular trimer structure. Evolution of discharging rings with increasing sample voltage *V*_s_ for various voltages: *V*_s_ = 0.54 V (**a**), 0.57 V (**b**), 0.62 V (**c**), 0.65 V (**d**), 0.69 V (**e**), 0.72V (**f**), 0.79 V (**g**), and 0.87 V (**h**). As *V*_s_ increases, the rings expand and intersect, and regions of NDC with distinct chiral pattern emerge in the center of the trimer. For the overlay with the molecular structure see Supplementary Fig. 5. **a'**–**h'**, The evolution of the discharge rings calculated using PME approach (in arb. units) for same sample voltages *V*_s_ as in the experiment. The effects of the TBTAP orbital structure were included in the calculation to simulate the chiral pattern in the experimental data. The scale bars are 1 nm.

The two-dimensional STS mapping of the TBTAP trimer provides a more direct insight into the discharge behavior. Figure 2a illustrates a d*I*/d*V* map of the trimer at *V*_s_ = 540 mV, revealing the presence of three distinct discharge rings corresponding to the three individual molecules (see Supplementary Fig. 6). Similar rings were previously observed in different nanostructures^8,13,37–40^. The evolution of discharging rings with increasing *V*_s_ from 570 to 870 mV is illustrated in Fig. 2b–h. As *V*_s_ increases, the rings expand in size and begin to overlap at *V*_s_ = 570 mV, leading to trillium-flower-shaped patterns. Upon further increase in the voltage, regions of NDC, holding a distinct chiral pattern, emerge near the center of the trimer. Similar flower-like characteristics were previously observed in WS_2_/WSe_2_ moiré lattices, although without any indication of NDC^8^.

In contrast to extended layered systems^7,8^, the finite size of our system allows for full theoretical description using a three-impurity Anderson model (TIAM). Details of the method, its implementation and the model parameters are summarized in the Supplementary Information. Each molecule is represented as a single correlated quantum level at an energy of −90 meV with respect to the surface chemical potential that interact via inter-site Coulomb (capacitive) coupling. The bias voltage is then incorporated to the model as the tip-position-dependent shift of the energy levels. The low-energy equilibrium physics of TIAM could be addressed by the numerical renormalization group (NRG) method. However, to gain insight into the behavior at sizable voltage, we performed a simulation utilizing the Pauli master equation (PME) approach. We assume here that the molecules are weakly coupled to the tip and the substrate (${\Gamma }_{{{\rm{t}}}} < {\Gamma }_{{{\rm{s}}}}\ll {k}_{B}{T}_{\exp }=0.22$ meV). Such value of coupling is significantly smaller than the equilibrium estimate from YSR states, ${\Gamma }_{{{\rm{s}}}}^{{{\rm{el}}}}=10$–20 meV ^14^. However, the latter is a low-energy equilibrium parameter fitted on energy scales  ~ 1 meV relevant for subgap spectroscopy, whereas the former is an effective coupling that governs non-equilibrium transport at biases of the order of hundreds of meV. A possible source of such reduction is electron-vibration coupling^41^, which is known to be substantial for TBTAP molecules on metallic surfaces^12^. Nevertheless, quantitative estimation of this effect is beyond the scope of this article. Such a small value of the coupling puts the system in the sequential tunneling regime, greatly simplifying the analysis of the experiment, as we can neglect any cotunneling effects.

The on-site Coulomb interaction strength *U* = 200 meV^12^ is large enough to prohibit double occupancy of a site. Furthermore, as we are not dealing with the effects of magnetic field or the Kondo physics, we consider the spin degree of freedom to be constrained. This significantly reduces the size of the configuration space of the molecular subsystem from 4^3^ = 64 to 2^3^ = 8 states. Other parameters of the model were fitted from a broad set of experimental data plotted in Supplementary Figs. 8 and 9. The results of the simulations are presented in Fig. 2a’–h’. The spatial distribution of the electron density associated with the SOMO in the TBTAP trimer was calculated using DFT (see Supplementary Fig. 4b) and incorporated into the simulation to elucidate the effects of the chirality, although it is not essential for the description of the charging patterns. The structure of the SOMO orbital was omitted for data presented in Supplementary Fig. 8 and replaced by a simple s-orbital, while all the main features, except the chiral pattern, are still present, showing the robustness of the effects. The slightly oval shape of the discharge rings in the experiment was introduced into the simulation by a finite quadrupole moment of the molecules. The almost perfect match between the experiment and the theory allows us to pinpoint physical parameters such as the value of the inter-site Coulomb coupling *W* = 50 meV, which is consistent with previous studies of TBTAP dimers^14^. The simulation also suggests a negligible value of the direct hopping between orbitals. As a result, charge rearrangement within the cluster is allowed only via the conductive substrate. This is in contrast to other suggested realizations of NDC in trimer structures, based on quantum interference effects^42,43^.

Low-energy YSR spectra were calculated using NRG for the model parameters inferred from the simulations of the charging rings and finite value of *U*, as, in contrast to the high-energy PME description, we cannot consider the spin to be irrelevant in this regime. The results show a doubly occupied ground state and a pair of slightly split YSR peaks, in agreement with the experiment, providing an additional check for the reliability of the model calculations. The simulations also suggest that the source of the YSR states splitting is the unequal coupling of the molecules to the substrate due to the presence of the scanning tip, which changes the height of the molecules through attractive force. Additional details about the YSR spectra are provided in the Supplementary Information.

### Negative differential conductance emerging from non-equilibrium occupancy of states

**Fig. 3:**
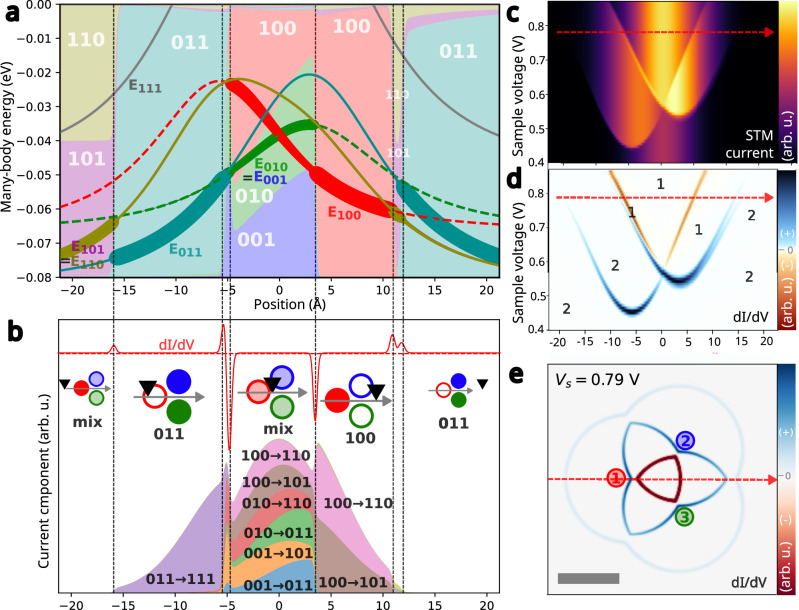
Simulation of the discharge rings. **a** Many-body state energy (lines) and non-equilibrium state occupation (shown both as thickness of lines, and as normalized stacked-plot to distinguish occupation of degenerate states, such as $| 001\left.\right\rangle$ and $| 010\left.\right\rangle$) calculated at sample voltage *V*_s_ = 0.79 V. The thickness of the line is proportional to the state occupation. The energy of the empty state defines the zero energy. **b** Absolute tunneling current decomposed to transitions between many-body states (stacked plot) and d*I*/d*V* (red line) at the same voltage as in panel a. Notice that currents are not normalized and therefore scale exponentially with distance between the tip and the sites. The five schematic drawings illustrate the character of molecular orbital occupancy (full/shaded/empty circles) for given position of the scanning tip (black triangle). **c** Two-dimensional tunneling current map with a cut at sample voltage *V*_s_ = 0.79 V depicted by red dashed arrow. **d** Differential conductance map for the same parameters as in (**c**). This result can be directly compared to the data in Fig. 1f. **e** Spatial map of d*I*/d*V* at *V*_s_ = 0.79 V. Red arrow marks the path along which the data in (**a**–**d**) are plotted. The scale bar is 1 nm.

In interacting multi-orbital systems, transport can generally involve quantum interference between different many-body tunneling amplitudes^44–46^. In the present model, however, the dots are coupled only capacitively and via a common metallic reservoir (surface), without direct tunneling. As a result, the dynamics at higher voltages can be interpreted in terms of transitions between charge configurations, with different transport channels contributing at the level of rates rather than phase-coherent amplitudes. To understand the origin of the charging rings and NDC regions observed in Fig. 2, we analyzed the individual components of the tunneling current obtained from PME calculations. The components, as well as their correspondence to the many-body state energies, are shown in Fig. 3a, b. Data are plotted for sample voltage *V*_s_ = 790 meV, along the line represented by red arrows in Fig. 3c–e, which passes directly over molecule 1 and between molecules 2 and 3. To keep the explanation simple, we assume a zero value of the quadrupole moment, which otherwise lifts the degeneracy of the on-site energies of molecules 2 and 3 (see Supplementary Fig. 10). Lines in Fig. 3a represent many-body state energies, while the thickness of the line marks the non-equilibrium state occupation. The occupation is also represented by the background stacked-plot to help distinguish occupation of degenerate states, such as $| 110\left.\right\rangle$ and $| 101\left.\right\rangle$ on the left side or $| 001\left.\right\rangle$ and $| 010\left.\right\rangle$ at the center. Figure 3b shows the corresponding tunneling current components together with the d*I*/d*V*. The currents are not normalized and therefore scale exponentially with the distance between the scanning tip and the site, until they suddenly change due to crossing of many-body levels, resulting in charge rearrangement within the cluster. The five schematic drawings then illustrate the site occupancy, which depends on the position of the scanning tip. The key insight lies in the significant capacitive coupling *W* ~ 50 meV, which makes charging the cluster with two electrons to −2*e* more energetically favorable than charging it with three electrons to −3*e*, despite the on-site energies being located 90 meV below the Fermi level of the substrate (i.e., the molecular LUMO being charged into SOMO). This results in a charge state, where typically two of the three molecular sites are occupied, and one remains empty. As the direct hopping between the sites is negligible, we consider the electrons to be localized on individual sites. Upon applying a sufficiently high bias voltage (sample voltage *V*_s_ = 0.6 V when the tip is at the center of the cluster), an additional electron is repelled from the cluster, leading to a net charge of −1*e*, meaning that only one site remains occupied.

The total charge of the cluster for different regions is marked in Fig. 3d, which shows the two-dimensional map of d*I*/d*V*, that corresponds to the experimental data plotted in Fig. 1f. In contrast to the intuitive interpretation of discharge rings in the d*I*/d*V* maps as indicating changes in total charge, many of the sharp transitions in site occupancy (manifesting as peaks in d*I*/d*V* spectra) actually correspond to intra-cluster charge rearrangements, i.e., electrons shifting between different molecular sites (through the conductive substrate) without change of total charge. This can be understood by analyzing the many-body ground state energies plotted in Fig. 3a. In a quasi-equilibrium regime (i.e., when the current from the tip is negligible and does not perturb the site occupancy), the system behavior is governed by the ground state alone. As the tip-position varies, the locally generated repulsive electric field destabilizes nearby occupied sites. In the language of many-body states, this causes the ground state to switch to a configuration where the site directly beneath the tip is empty, opening up a tunneling channel from the tip to that site.

Counterintuitively, already the outer ring visible as the blue arc on the left side of Fig. 3e, respectively, the blue parabola on the left side of Fig. 3d is not related to a change of total charge, but to rearrangement within the doubly occupied cluster (namely from a linear combination of $| 101\left.\right\rangle$ and $| 110\left.\right\rangle$ on the left to $| 011\left.\right\rangle$ on the right). The NDC arises instead in the central region between the excited-state crossings, where the energies of singly occupied excited states $| 100\left.\right\rangle$, $| 010\left.\right\rangle$, and $| 001\left.\right\rangle$ become lower than doubly occupied excited states (e.g., $| 101\left.\right\rangle$ and $| 110\left.\right\rangle$). Due to the absence of inter-site hopping in our model, these singly occupied states cannot transit from one state to another by single-electron tunneling processes, leading to non-equilibrium occupancy distributions. Consequently, the system enters a mixed state in which the occupancy is approximately evenly distributed between the sites.

The resulting suppression of conductance in this central region can be understood in terms of distance dependence of tunneling between the tip and sites, which is in our simplified case modeled as ${t}_{ti}\propto \exp (-\beta | {{{\bf{r}}}}_{i}-{{{\bf{r}}}}_{t}| )$, where **r**_*i*_ is site position, **r**_*t*_ tip position and *β* is a decay constant. In contrast to the quasi-equilibrium regime, where the tip field pushes the electron out of the nearest site, which also has the highest tunneling amplitude, in the non-equilibrium regime, a large fraction of the occupation probability is wasted in states where the unoccupied site is far from the tip, leading to ineffective tunneling. In reality, the angular dependence of tunneling matrix elements considering symmetry of the relevant molecular orbital suppresses the current for some tip positions due to nodes of the orbitals, reducing the NDC region and giving rise to the chiral pattern observed in both experiment and simulation (Fig. 2).

Direct frequency-shift spectroscopy, *Δf*(*V*), as measured in non-contact atomic force microscopy, would provide additional support for distinguishing charged and neutral molecular states within the trimer. In the present work, however, the proposed charge configurations are inferred from the combined evidence of the spatially resolved *dI*/*dV* signatures, the low-energy YSR spectra and the many-body transport calculations, consistent with our previous observation that charged TBTAP molecules on Pb(111) exhibit a characteristic *Δf*(*V*) dip associated with tip-induced discharge via capacitive coupling^47^.

### Triangular hexamer assemblies

Another type of triangular structure, composed of six TBTAP molecules, was constructed using the same lateral manipulation technique (for details on the geometry, see Supplementary Fig. 13a). The assembly can be manipulated across the Pb(111) surface, showing high structural stability (Supplementary Fig. 14). The hexamer structure introduces two distinct molecular environments. The three inner TBTAP molecules at the core of the assembly form a trimer similar to the one discussed in the previous section; however, the three outer molecules modify its local electrostatic environment. Supplementary Fig. 13b displays the d*I*/d*V* spectra acquired from the inner and outer TBTAP molecules, marked by colored dots. In particular, charging dips are observed for the three inner molecules for sample voltages between −300 and −400 mV, whereas the outer molecules do not display significant features within this voltage range. The spectra suggest that the inner molecules are initially at *V*_s_ = 0 in neutral TBTAP^0^ state, while the outer molecules are charged. This is in agreement with our previous results on short chains of TBTAP molecules, which show that the molecule can lose its radical nature due to the presence of an additional electrostatic field from neighboring molecules^14^. In addition, YSR spectroscopy (Supplementary Fig. 13d) further proves the presence of these two charge states in the hexamer.

**Fig. 4:**
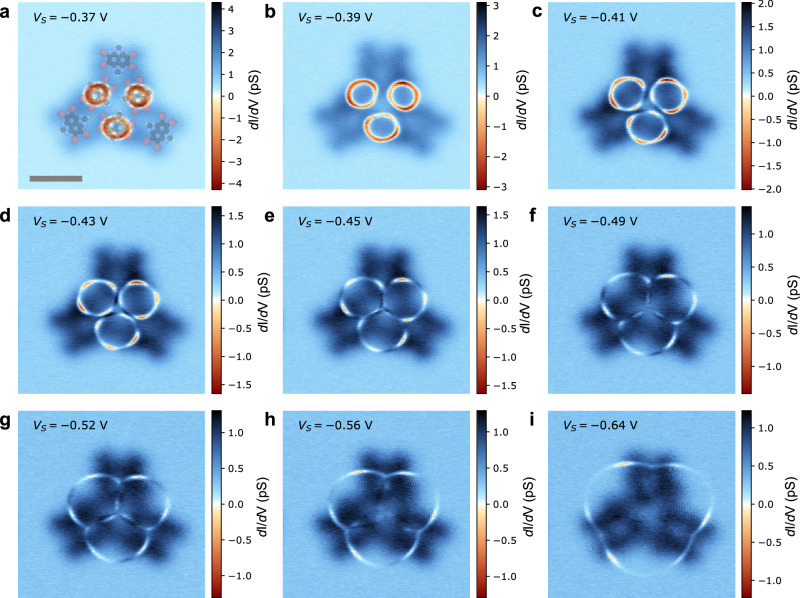
Charging rings in the hexamer structure. The evolution of charging rings in the hexamer assembly with decreasing sample voltage for *V*_s_ = −0.37 V (**a**), −0.39 V (**b**), −0.41 V (**c**), −0.43 V (**d**), −0.45 V (**e**), −0.49 V (**f**), −0.52 V (**g**), −0.56 V (**h**), and −0.64 V (**i**). Regions of NDC form around the inner molecules with decreasing negative voltage, as a result of the onset of the Coulomb blockade, intersecting at *V*_s_ = − 0.45 V. **a** Includes an overlaid molecular structure to indicate the positions of the molecules in the hexamer. The scale bar is 1 nm.

Figure 4a–i illustrates the evolution of the charging rings in d*I*/d*V* spectra with *V*_s_ decreasing from −0.37 V to −0.64 V (for the corresponding current maps see Supplementary Fig. 15). The charging rings around the inner molecules in the hexamer increase in size and intersect at *V*_s_ = −0.45 V. The rings show a distinct four-lobe pattern, similar to the trimer case, as a result of the TBTAP orbital structure. Furthermore, just before the rings intersect, a chiral pattern of positive d*I*/d*V* emerges near the center of the structure for sample voltages around −0.43 V. However, in contrast to the trimer, no additional patterns emerge at the center of the structure after the rings intersect.

We did not perform a thorough theoretical analysis of the hexamer system, as the behavior is more monotonous and less surprising in comparison to the trimer. The additional electrostatic field from the charged outer molecules shifts the local energy level of the inner molecules above the substrate chemical potential, leaving them unoccupied at zero bias. At negative sample voltage (i.e., positive tip voltage), the electric field of the tip pushes these states below the substrate’s Fermi level, causing them to become occupied. This event gives rise to the charging rings observed around each inner molecule, which exhibit NDC at all voltages beyond the charging threshold. The drop in STM current can be understood within the framework observed in TCNQ layers^7^. On the one hand, the charging of an inner molecule opens an additional, sequential tunneling channel via its now-occupied orbital. On the other hand, the local negative charge simultaneously increases the tunneling barrier for all tunneling paths (i.e. non-resonant current from the substrate and from the outer molecules). The net effect is a decrease in total conductance. Besides the charging rings observed over the inner molecules, one would also expect discharging rings to form around the outer molecules at a positive sample voltage. This was not observed in the experiment as the voltage needed to discharge the outer molecules might be larger than 1.2 V, which could damage the sample.

To conclude, we have demonstrated that a cluster of three molecules, assembled with atomic precision, exhibits both positive and negative differential conductance at positive sample voltages arising from correlated intra-cluster charge dynamics. Thanks to the finite and tractable Hilbert space, it is possible to fully simulate its non-equilibrium dynamics and rationalize the observed conductance features using the PME approach. In particular, we identify a regime where the non-equilibrium occupation of excited states leads to a suppression of tunneling current, resulting in NDC. The analysis also reveals that the prominent conductance peaks observed in d*I*/d*V* spectra are not necessarily associated with changes in the total charge, but often originate from rearrangements of charge between molecules. These effects are further modulated by orbital symmetry, giving rise to the observed chiral patterns. The observation of similar phenomena in a more complex hexamer structure further confirms the robustness of these effects.

## Methods

### Lateral manipulation

To manipulate individual TBTAP molecules on the Pb(111) surface, we utilized a lateral manipulation technique that is widely applied for moving molecules across metallic substrates. The procedure began by positioning the STM tip directly above the selected molecule using standard imaging conditions (*I* = 100 pA, *V*_s_ = 100 mV). Next, the tip was brought closer to the molecule by adjusting the tunneling conditions to *I* = 3 nA and *V*_s_ = 3 mV, with the feedback loop turned off. While maintaining this configuration, the tip was laterally translated at a velocity of 200 pm/s, effectively dragging the molecule toward the desired site. Once the final position was reached, the feedback loop was re-engaged, and the initial scanning parameters were restored (*I* = 100 pA, *V*_s_ = 100 mV).

### STM/STS experiments

STM and STS experiments were performed with a low-temperature (2.6 K) Joule-Thomson STM/AFM microscope (purchased from Omicron GmbH) in ultra-high vacuum (UHV) of ≈10^−10^ mbar operated with Nanonis RC5e electronics. Differential conductance (d*I*/d*V*) spectra were recorded with an internal Nanonis lock-in amplifier using modulation amplitudes indicated in the figure captions. The measurements were performed with a pure Pb tip to enhance the energy resolution beyond the thermal limit.

### DFT calculations

DFT was used to calculate the equilibrium position of a TBTAP trimer adsorbed onto Pb(111) surface. Calculations were carried out using Turbomole 7.5.1^48^ridft code utilizing the def2-SV(P) (double-zeta) basis set and B3-LYP exchange-correlation functional with D3(BJ) dispersion correction. The Pb surface was modeled as a slab of three atomic layers. The system was charged by three electrons to simulate charge transfer to the molecules.

### Transport calculations

Simulations of the tunneling spectra for the molecular trimer were performed using a code developed by us, based on the PME approach. The implementation of the master equations was derived from the QmeQ package^49,50^, with an integrated model of the electrostatic field of the tip, SPM image simulations, and a graphical user interface. In these calculations, we neglected the superconducting nature of the substrate, as it does not have any effect on the discharge rings. The system was mapped on the three-impurity Anderson model with two metallic leads, the substrate and the scanning tip. The details of the model and the PME approach are described in the Supplementary Information. Some of the results of our code were verified using the QmeQ package, showing perfect agreement. The code is available on GitHub^51^. We hope this software will support future studies of similar systems by other experimental groups.

### Deconvolution of the YSR tunneling spectra

A deconvolution procedure was utilized to obtain the low-energy, YSR spectra from the d*I*/d*V* data. We used the maximum entropy method implemented in a modified ana_cont package^52^. Additional details on the procedure are presented in ref. ^14^. A flat default model of the same width as the input data was used. The optimal value of the hyperparameter *α* was obtained using the chi2kink method^53^. The scanning tip parameters were fitted by a two-step procedure. First, the sum of the gap widths of the tip and the sample *Δ*_t_ + *Δ*_s_ = 2.62 meV was fitted from the tunneling spectra measured on a bare Pb surface. Then, the difference between the gap widths *Δ*_t_ − *Δ*_s_ was fitted from the positions of the thermal images, which are well pronounced in the tunneling spectra due to the higher experimental temperature ${T}_{\exp }=2.6$ K. The results read *Δ*_t_ = 1.35 meV and *Δ*_s_ = 1.27 meV. The value of the broadening parameter obtained by the initial fit is *γ*_s_ = *γ*_t_ = 0.03 meV. The modified ana_cont code is available on GitHub^54^.

### NRG calculations of the YSR spectra

NRG calculations were performed using the open-source NRG Ljubljana package^55^. Following previous results^14^, we fixed the half-bandwidth of the conduction band to 1 eV (unity in the code) and the superconducting gap to *Δ* = 1.3 meV. The charging energy was set to *U* = 200 meV, in line with earlier studies^12^. We focus on the most strongly correlated system with *ζ* = 1 (see the discussion below Eq. (5) in the Supplementary Information), which enables single-channel calculations. For reproducibility, we provide here the specific parameters used in our calculations; detailed descriptions of these parameters are available in the package manual. The subgap spectrum and related quantities were obtained using the following settings: lambda = 2, symtype = SPSU2, keepenergy = 10, keep = 4000, and keepmin = 4000. The impurity spectral functions were calculated using *z*-averaging with *N*_*z*_ = 40 and the modified log-Gaussian kernel broadening (smooth = newsc), allowing for different broadening values within the superconducting gap (*ω*_0_ = 3 × 10^−5^) and above it (*α* = 0.05).

## Supplementary information

Supplementary Information

Transparent Peer Review file

## Data Availability

All the data supporting the findings of this study are available on Zenodo^56^. The data are also available from the corresponding authors upon request.

## Code availability

The code for the simulations of tunneling spectra is available on GitHub^51^. The version of the code that was used in this work is also included in the Zenodo repository^56^.
