# Supplementary material for: Non-equilibrium correlated electron dynamics in triangular molecular assemblies
Source: Nat Commun. 2026 Jul 8;17:8406. doi: 10.1038/s41467-026-75051-3 (PMC13473136; doi:10.1038/s41467-026-75051-3)
Supplement: Supplementary file 1 [file 41467_2026_75051_MOESM1_ESM.pdf]

Supplemental Information: Non-Equilibrium Correlated Electron Dynamics in Triangular Molecular Assemblies

Chao Li*,[1,2] Vladislav Pokorný*,[3] Prokop Hapala*,[3] Martin Žonda,[4] Thilo Glatzel,[2] Ping Zhou,[5] Silvio Decurtins,[5] Shi-Xia Liu,[5] Fengqi Song,[1] Ernst Meyer,[2] and Rémy Pawlak[2]

[1]*Institute of Atom Manufacturing, Nanjing University, Nanjing 210089, China.*
[2]*Department of Physics, University of Basel, Klingelbergstrasse 82, 4056 Basel, Switzerland.*
[3]*Institute of Physics (FZU), Czech Academy of Sciences, Na Slovance 2, 182 00 Prague 8, Czech Republic.*
[4]*Department of Condensed Matter Physics, Faculty of Mathematics and Physics, Charles University, Ke Karlovu 5, 121 16 Prague 2, Czech Republic.*
[5]*Department of Chemistry, Biochemistry and Pharmaceutical Sciences, W. Inäbnit Laboratory for Molecular Quantum Materials and WSS Research Centre for Molecular Quantum Systems, University of Bern, Freiestrasse 3, 3012 Bern, Switzerland.*

Emails: chao.li@unibas.ch; pokornyv@fzu.cz; shi-xia.liu@unibe.ch

SUPERCONDUCTING THREE-IMPURITY ANDERSON MODEL

In all our calculations, including the analysis of the YSR states, we represent the TBTAP trimer using the superconducting three-impurity Anderson model (TIAM) with triangular geometry (Supplementary Fig. 1). The Hamiltonian reads

$$\mathscr{H} = \mathscr{H}_{\rm imp} + \mathscr{H}_{\rm lead} + \sum_{i=1}^{3} \mathscr{H}^{(i)}_{\rm hyb}. \tag{1}$$

The three impurities are described as

$$\mathscr{H}_{\rm imp} = \sum_{i\sigma} \left[\varepsilon_i(\mathbf{r}) n_{i\sigma} + U_i n_{i\uparrow} n_{i\downarrow}\right] + \sum_{i,j>i} W_{ij} n_i n_j - \sum_{i,j>i,\sigma} t_{ij} \left(d^\dagger_{i\sigma} d_{j\sigma} + \text{H.c.}\right). \tag{2}$$

Here $d^\dagger_{i\sigma}$ creates an electron with energy $\varepsilon_i(\mathbf{r}) = \varepsilon_{id} + \nu_i(\mathbf{r})$ and spin σ on the impurity $i = 1, 2, 3$, ε_{id} is the impurity energy level in absence of the scanning tip, $\nu_i(\mathbf{r})$ is the energy shift due to the presence of the scanning tip at position $\mathbf{r}$, U_i is the local, on-site Coulomb interaction, W_{ij} is the inter-site capacitive coupling between impurities i and j, t_{ij} is the direct hopping between impurity orbitals and $n_{i\sigma} = d^\dagger_{i\sigma} d_{i\sigma}$ and $n_i = n_{i\uparrow} + n_{i\downarrow}$ are the particle number operators. The superconducting substrate (s) and the scanning tip (t) are described by

$$\mathscr{H}_{\rm lead} = \sum_{\alpha\mathbf{k}\sigma} \varepsilon_{\alpha\mathbf{k}} c^\dagger_{\alpha\mathbf{k}\sigma} c_{\alpha\mathbf{k}\sigma} - \sum_{\alpha\mathbf{k}} \left(\Delta_\alpha c^\dagger_{\alpha\mathbf{k}\uparrow} c^\dagger_{\alpha-\mathbf{k}\downarrow} + \text{H.c.}\right), \tag{3}$$

where $c^\dagger_{\alpha\mathbf{k}\sigma}$ creates an electron in lead $\alpha = s, t$ with energy $\varepsilon_{\alpha\mathbf{k}}$ and spin σ and Δ_α is the superconducting order parameter.

The coupling between the impurities and the substrate and the scanning tip is described by

$$\mathscr{H}^{(i)}_{\rm hyb} = \sum_{\mathbf{k}\sigma} \left(t_{\text{s}i\mathbf{k}} c^\dagger_{\text{s}\mathbf{k}\sigma} d_{i\sigma} + t_{\text{t}i\mathbf{k}}(\mathbf{r}) c^\dagger_{\text{t}\mathbf{k}\sigma} d_{i\sigma} + \text{H.c.}\right), \tag{4}$$

where $t_{\alpha i\mathbf{k}}$ is the tunneling matrix element between lead α and impurity i.

In all our calculations, we make the following assumptions: (1) Site-independent value of the capacitive coupling $W_{ij} = W < U$ and hopping $t_{ij} = t$. (2) Same superconducting gap of the scanning tip and the substrate $\Delta_{\rm s} = \Delta_{\rm t} \equiv \Delta$.

---

* These authors contributed equally to this work.

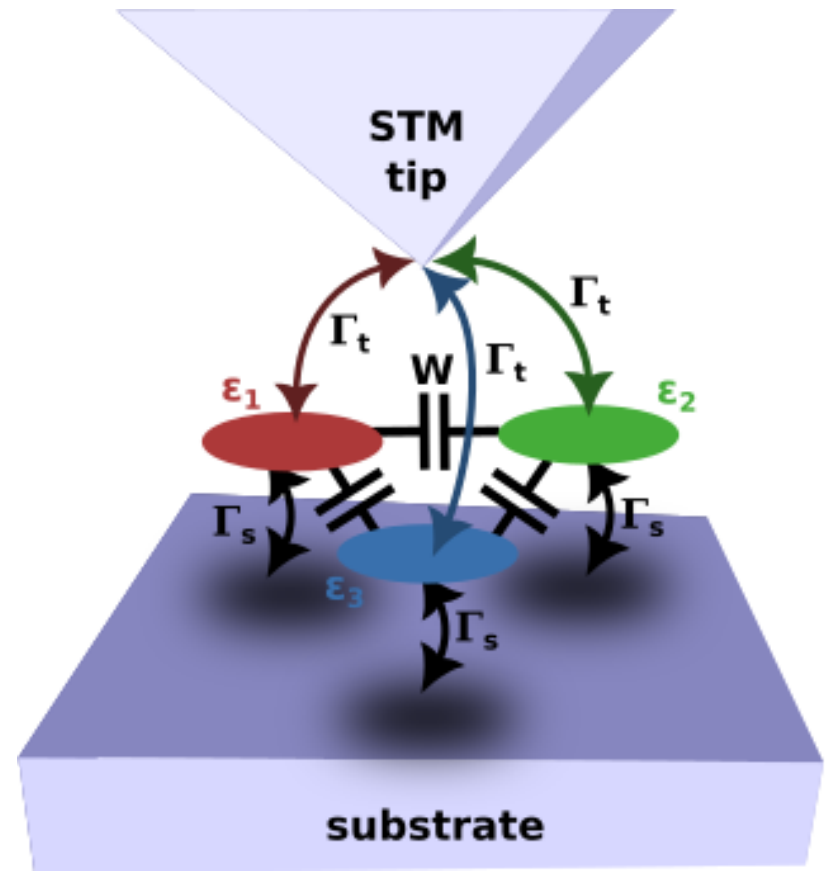

Supplementary Figure 1. | **Schematics of the setup.** Three molecules in triangular geometry (red, green and blue discs) are represented by single energy levels ε_i and interact via inter-site capacitive coupling W. The sites are coupled to the substrate (scanning tip) via coupling strength Γ_s (Γ_t). The direct hopping t_{ij} is omitted from the schematics for simplicity.

(3) Same value of the coupling to the substrate, $t_{\mathrm{s}i\mathbf{k}} = t_{\mathrm{s}\mathbf{k}}$ while $t_{\mathrm{t}i\mathbf{k}}$ depends on the actual position of the scanning tip, as explained below. (4) tunneling rates

$$\Gamma_{\alpha ij}(\omega) = \pi \sum_{\mathbf{k}} t^*_{\alpha i\mathbf{k}} t_{\alpha j\mathbf{k}} \delta(\omega - \varepsilon_{\alpha\mathbf{k}}) \equiv \Gamma_{\alpha ij} \tag{5}$$

are energy-independent. From that we can define momentum-independent tunnel matrix elements $t_{\alpha i} = \sqrt{\Gamma_{\alpha ii}/(\pi\rho_{0\alpha})}$, where $\rho_{0\alpha}$ is the Fermi-energy value of the density of the states (DOS) of the lead α, which we consider to be constant, $\rho_\alpha(\omega) = \Theta(D_\alpha^2 - \omega^2)/(2D_\alpha)$, where D_α is the half-bandwidth. This leads to $\rho_{0\alpha} = 1/(2D_\alpha)$. We denote the local tunneling rates $\Gamma_{\mathrm{s}ii} \equiv \Gamma_\mathrm{s}$ and $\Gamma_{\mathrm{t}ii} \equiv \Gamma_{\mathrm{t}i}$. (5) For the non-local ($i \neq j$) hybridization terms we assume that $\Gamma_{\mathrm{t}ij} = 0$ and $\Gamma_{\mathrm{s}ij} = \zeta\Gamma_\mathrm{s}$, where $0 \leq \zeta \leq 1$ is a parameter that indirectly encodes the distance between the impurities. (6) Both leads are thermalized according to a Fermi–Dirac distribution $f_\alpha(\omega) = \big(\exp[(\omega - \mu_\alpha)/k_B T_\mathrm{exp}] + 1\big)^{-1}$, where μ_α is the chemical potential of lead α and T_exp is the experimental temperature.

TIP ELECTROSTATICS

The influence of the electrostatic field of the tip on molecular energy levels $\varepsilon_i(\mathbf{r})$ is the key ingredient in simulating the charging rings. The challenge is that the exact shape of the electrostatic field in STM experiments is unknown, as it depends on the uncontrollable geometry of the tip. A variety of models for AFM/STM tips exist in the literature (e.g., spherical, conical, or pyramidal shapes) [1–4]. For this reason, we developed our own electrostatic model suitable for $\mathrm{d}I/\mathrm{d}V$ simulations of organic molecules on flat substrates. In our model, the nanojunction is treated as a parallel-plate capacitor defined by two conducting planes at heights z_C and z_S and biased by a voltage V_s. The molecules are placed at $z = 0$, with the substrate (i.e. the second capacitor plate) located at $z_\mathrm{S} < 0$ below. A single spherical adatom of radius r_tip is placed at height z_tip above the molecules that contributes an additional radial Coulomb-like potential. The bottom capacitor plate is grounded, and this surface need not coincide with the top atomic layer of the substrate. Instead, z_S is treated as a fitting parameter to match experimental features (particularly the evolution of the size of the charging rings with increasing V_s). This surface also acts as an electrostatic mirror for the molecular charge and multipole moments. We found that both the capacitor plate and the electrostatic mirror are essential to reproduce the exact shape of the charging rings. The molecular orbitals interact with the tip field via monopole charge $1e^-$. To account for the shape of the molecule, we also include a quadrupole moment Q_{xx} aligned along the molecular long axis, which is fitted to reproduce the experimentally observed ellipticity of the charging rings. Due to molecular symmetry, the dipole and other quadrupolar components vanish. Both monopole and quadrupole moments are mirrored across the substrate at $z = -2z_S$.

TIP COUPLING

Tunneling between the tip and the sample is, naturally, an essential component of STM/STS simulations. In typical quasi-equilibrium situations, when the molecule is strongly coupled to the substrate and its energy levels are filled up to the substrate's Fermi level, the tunneling current I can be calculated using the Fermi golden rule,

$$I(V) \propto \int dE \sum_i |t_{\mathrm{t}i}|^2 \left[f_{\mathrm{t}}(E-eV) - f_{\mathrm{s}}(E)\right], \tag{6}$$

where f_{t} and f_{s} are the Fermi functions of the tip and substrate, respectively, and $t_{\mathrm{t}i} = \langle\psi_i|H'|\psi_{\mathrm{t}}\rangle$ is the tunneling matrix element between the molecular orbitals ψ_i (within the tunneling window) and tip states ψ_{t}. The perturbation H' originates from the potential of one subsystem acting on the other. In practice, it is often approximated as constant, simplifying the tunneling matrix to an overlap, $t_{\mathrm{t}i} \approx C\langle\psi_i|\psi_{\mathrm{t}}\rangle$. For atomically sharp tips, the tip wavefunction ψ_{t} is usually modeled as an s-orbital. More sophisticated models are generally unfeasible due to limited knowledge of the atomic structure of the tip.

In previous work, we implemented the `PPSTM` code [5] for quasi-equilibrium STM/STS simulations, where tunneling matrix elements were evaluated using Chen's rules in a atomic basis [6]. Later, we simplified and optimized this scheme using 2D or 3D convolution of molecular orbitals projected onto a grid, which was used for simulations of the light-STM technique with the `PPAFM` code [7, 8]. This approach leverages the convolution theorem and fast Fourier transform (FFT) to efficiently compute entire 2D images or 3D stacks in one pass. In the present molecular system, the situation is more complex because the system is out of equilibrium and the occupancy of molecular states must be determined by solving the master equations. However, tunneling rates into and out of the molecular states, which serve as input parameters to the ME, are still calculated using the same tunneling matrix elements based on the Fermi golden rule assuming s-orbital symmetry of the tip wavefunction ψ_{t}.

We found that orbital symmetry, reflected in the elements of the tunneling matrix, is essential to reproduce intensity modulation (four lobes separated by nodal planes) and the chiral features of the d$I(x,y)$/dV maps of the TBTAP trimer. This approach was used to obtain results in Fig. 2 in the main text. However, for simplicity, most of our simulations were performed using a basic model of exponentially decaying hopping, $t_{\mathrm{t}i} = \exp(-\beta|\mathbf{r}_i - \mathbf{r}_{\mathrm{t}}|)$, where $\mathbf{r}_i$ is the position of the i-th molecule (the center of mass of the relevant molecular orbital), $\mathbf{r}_{\mathrm{t}}$ is the position of the tip apex, and β is a tunable parameter. This corresponds to modeling the orbital ψ_i as a spherically symmetric s-function. Despite its simplicity, this model is sufficient to qualitatively reproduce all the characteristics of the charging rings, including those associated with NDC, as presented in Supplementary Fig. 8.

PAULI MASTER EQUATIONS

We applied the approximate master equation approach [9] to the TIAM without superconducting pairing in the Coulomb blockade regime to obtain the current from the scanning tip to the molecular trimer. Neglecting superconducting correlations here is justified, as the relevant features occur at large sample bias ($V > 400\,\mathrm{meV}$), well exceeding the superconducting gap ($\Delta \approx 1.3\,\mathrm{meV}$). Here we assume that the time between two tunneling events is the largest time scale, i.e. the sequential tunneling regime. We also assumed infinite value of the local Coulomb interaction U which makes the energy of the doubly occupied states prohibitively large. This assumption largely simplifies the calculations, as it reduces the dimension of the configurational space from 64 to 8. The value of the molecular energy level in the absence of the scanning tip was fixed to $\varepsilon_{id} = -90$ meV as assumed from the data in Supplementary Fig. 3. The actual scanning-tip-position dependent values of of the molecular energy levels $\varepsilon_i(\mathbf{r})$ and the tip-sample tunneling amplitudes $t_{\mathrm{t}i}(\mathbf{r})$ were calculated using the approach from previous sections.

We diagonalized Hamiltonian (2) to get the many-body eigenstates (note that this step is not necessary in case of $t = 0$ model without direct hopping), $\mathscr{H}_{\mathrm{imp}} = \sum_a E_a|a\rangle\langle a|$ and the many-body tunneling amplitudes $T^{\alpha}_{ba} = \sum_i V_{\alpha i}\langle b|d^{\dagger}_i|a\rangle$, $\alpha = s, t$. To obtain the current through the device we use the simplest PME approach, in which the coherences are not included and only state populations P_α (diagonal elements of the reduced density matrix) are considered. The probabilities are given by the kinetic (master) equations [9]

$$\begin{aligned}\frac{\partial P_b}{\partial t} &= \sum_{\alpha,a}\left[P_a\Gamma^{\alpha}_{a\to b} f(x^{\alpha}_{a,b}) - P_b\Gamma^{\alpha}_{b\to a} f(-x^{\alpha}_{a,b})\right] \\ &+ \sum_{\alpha,c}\left[P_c\Gamma^{\alpha}_{c\to b} f(x^{\alpha}_{c,b}) - P_b\Gamma^{\alpha}_{b\to c} f(-x^{\alpha}_{c,b})\right]\end{aligned} \tag{7}$$

where $x^{\alpha}_{a,b} = (E_a - E_b - \mu_\alpha)/k_B T_\alpha$ and $\Gamma^{\alpha}_{a\to b} = 2\pi\rho_{0\alpha} T^{\alpha}_{ba} T^{\alpha}_{ab} = \Gamma^{\alpha}_{b\to a}$. Here we use the notation in which the particle numbers follow $N_a = N_b - 1$ and $N_c = N_b + 1$. These equations are solved in the stationary limit $\partial P_b/\partial t = 0$ with the normalization condition $\sum_b P_b = 1$.

The steady state current from lead α to the molecular trimer is then given by

$$I_\alpha = \frac{e}{\hbar}\sum_{ab}\left[P_a \Gamma^{\alpha}_{a\to b} f(x^{\alpha}_{a,b}) - P_b \Gamma^{\alpha}_{b\to a} f(-x^{\alpha}_{a,b})\right]. \tag{8}$$

We chose a small value of the substrate coupling strength $\Gamma_{\rm s} \sim 0.01$ meV $\ll k_B T_{\rm exp}$ to stay in the sequential tunneling regime and the validity regime of the PME. This value is much smaller than what can be inferred from the experimental data for TBTAP on Pb(111), $\Gamma_{\rm s,exp} = 10 - 20$ meV [10]. A plausible source of reduction are some additional non-equilibrium renormalization mechanisms, in particular strong electron-vibration coupling, as discussed briefly in the main text. In addition, the size and shape of the discharge rings do not depend on the value of Γ, but rather on the electrostatic details governed by the local energy levels $\varepsilon_i(\mathbf{r})$ and the capacitive coupling W. Only the width of the discharge ring is proportional to $\Gamma_{\rm s}$ and $\Gamma_{\rm t}$, while in our calculation it is given solely by the temperature $k_B T_{\rm exp}$.

We obtained the parameters of the model by fitting the experimental data plotted in Supplementary Fig. 8. The fitted parameters of the electrostatic model read $r_{\rm tip} = 0.3$ nm, $z_{\rm tip} = 0.6$ nm, $z_{\rm C} = 2$ nm and $z_{\rm S} = -0.09$ nm. Note that the position of the electrostatic mirror $z_{\rm S}$ does not match the distance of the Pb(111) surface from the molecules which is around 0.3 nm. The fitted value of the inter-site Coulomb coupling reads $W = 50$ meV, in agreement with the previous fit of the YSR spectra of TBTAP dimers [10]. We also compared the result for infinite value of U with the results for $U = 200$ meV calculated using the `QmeQ` package [11]. The comparison plotted in Supplementary Fig. 7 shows little or no differences, which justifies the previous assumption. The best fit of the experimental data was obtained for the zero value of inter-site hopping t. This further simplifies the analysis, as in the absence of direct hopping, the eigenstates of $\mathscr{H}_{\rm imp}$ are just the basis states that we label

$$|000\rangle,\quad |100\rangle,\quad |010\rangle,\quad |001\rangle,\quad |011\rangle,\quad |101\rangle,\quad |110\rangle,\quad |111\rangle, \tag{9}$$

where $|100\rangle = d_1^\dagger|000\rangle$, $|110\rangle = d_2^\dagger d_1^\dagger|000\rangle$ etc.

The energies of the states for sample voltage $V_{\rm s} = 0$ read 0, ε_{id}, $2\varepsilon_{id} + W$ and $3\varepsilon_{id} + 3W$ for empty, singly occupied, doubly occupied and triply occupied states, respectively. From this we obtain the value of the capacitive coupling W at which the ground state (GS) changes from triply to doubly occupied, $W = -\varepsilon_{id}/2$. For $W = 50$ meV and $\varepsilon_{id} = -90$ meV, the GS is doubly occupied, as noted in Figs. 1e-f and Fig. 3d in the main text. The many-body energies of the isolated cluster for two values of the sample voltage $V_{\rm s} = 0.51$ V and 0.79 V, which also include the effects of the quadrupole moment, are plotted in Supplementary Fig. 10. Here, the energy of the empty state $|000\rangle$ marks the energy zero. The ground state for $V_{\rm s} = 0.51$ V is always doubly occupied, illustrating the situation where crossing the discharge ring is not connected with a change of the total charge, but rather a redistribution of the charge inside the cluster. The triply occupied state always lies at higher energies.

YSR SPECTRA

The spinful character of the TBTAP$^{\bullet-}$ anion deposited on the superconducting surface gives rise to distinct YSR features in the spectra measured at low energies $eV_{\rm s} \sim \Delta_{\rm s}$, revealing additional details about the charge state of the TBTAP assemblies. The measurements were performed with a pure Pb tip to enhance the energy resolution beyond the thermal limit. As a result, the tunneling spectra are a convolution of the tip and the sample DOS. A deconvolution procedure based on the maximum entropy method (see the Methods section) was used to obtain the sample DOS. The results for three positions of the scanning tip presented in Supplementary Fig. 11a-f show two pairs of well-developed YSR peaks at sample voltages $V_{\rm s} = \pm 1.85$ mV and ± 1.95 mV that correspond to YSR energies $E_{YSR} = eV_{\rm s} - \Delta_{\rm t} = 0.50$ and 0.60 meV. The faint features observed at $V_{\rm s} = \pm 0.75$ mV are thermal replicas of the YSR peaks that appear due to the finite experimental temperature $T_{\rm exp} = 2.6$ K. The spatial map of $\mathrm{d}I/\mathrm{d}V$ at $V_{\rm s} = \pm 1.8$ mV in Supplementary Fig. 11i reveals a distinct chiral pattern of the YSR states, similar to the structures observed in the high-energy NDC features presented in Fig. 2 in the main text.

We employed the superconducting TIAM to model the YSR states. The model was solved using NRG (see the Methods section) with parameters similar to those used in the discharge-ring simulations: $U = 200$ meV, $W = 50$ meV, and $t = 0$. The coupling to the scanning tip, $\Gamma_{\rm t}$, was neglected, while a realistic substrate coupling of $\Gamma_{{\rm s}i} = \Gamma_i = 20$meV

was used [10], with $\zeta = 1$. The influence of the tip is addressed later. For simplicity, we assumed identical local energies on all sites, $\varepsilon_{id} = \varepsilon$.

Supplementary Fig. 12a shows the evolution of the sub-gap many-body spectrum calculated using NRG with respect to the GS energy E_0, as a function of the on-site energy ε. The data show a pair of quantum phase transitions, first at $\varepsilon = -58.5$ meV between the singlet and the doublet GS, and a second at $\varepsilon = -111.5$ meV between the doublet and the triplet GS (compare to the exact result for vanishing Γ, which predicts the transition at $\varepsilon = -2W = -100$ meV). The average occupation of the trimer is plotted in Supplementary Fig. 12b. The result is in agreement with the PME calculation that also predicts the charge $-2e$ for small values of the sample voltage V_s and realistic values of the local energy $\varepsilon \sim -100$ meV. The YSR states in this region correspond to doublet-singlet transitions (orange dots in panel a). In the experiment, the YSR states are split by approx. 0.1 meV. Such a splitting can be the result of an asymmetry in the trimer structure, induced by the attractive force between the scanning tip and the molecules, which decreases the surface coupling. We simulate the effect of the tip by modifying the coupling Γ_1 of one molecule. The results in Fig. 12c indeed show splitting of the many-body energies, which are otherwise degenerate pairs of doublets and singlets. As the YSR states correspond to the allowed transitions between the many-body states (e.g., doublet-singlet), this effect is a possible cause of the splitting observed in the experiment. Supplementary Fig. 11g shows the DOS calculated using NRG for two cases, symmetric case with $\Gamma_1 = \Gamma_2 = \Gamma_3 = 20$ meV (red line) and slightly asymmetric case for $\Gamma_1 = 19.5$ meV (blue line). The small asymmetry in the coupling leads to splitting of the YSR states as predicted, which is in agreement with the experimental result.

YSR spectroscopy was also performed on a hexamer assembly, Supplementary Fig. 13d, revealing a YSR peak on the outer three molecules. No YSR features were observed on the inner three molecules, except a faint peak that can be attributed to the tail of a YSR state from an outer molecule. This provides further proof of the loss of spinful radical nature of the inner molecules, as discussed in the main text.

NRG IMPLEMENTATION DETAILS

The NRG calculations were performed using the open-source `NRG Ljubljana` package [12]. The justification of physical parameters are discussed in the Methods section of the main text. Here, for reproducibility, we provide the explicit model definition as implemented within the SNEG/NRG Ljubljana framework. For simplicity, we focus on the regime of strongest correlations through substrate $\zeta = 1$, which allows for a single-channel formulation. The single-channel model is defined as:

```
def1ch[3]; (* three impurities, named d, a and b *)

H1 = delta1 number[d[]] + U1/2 pow[number[d[]]-1, 2];
H2 = delta2 number[a[]] + U2/2 pow[number[a[]]-1, 2];
H3 = delta3 number[b[]] + U3/2 pow[number[b[]]-1, 2];

H12 = -t12 hop[d[], a[]];
H13 = -t12 hop[d[], b[]];
H23 = -t12 hop[a[], b[]];

Hc1 = gammaPol1 hop[f[0], d[]];
Hc2 = gammaPol2 hop[f[0], a[]];
Hc3 = gammaPol3 hop[f[0], b[]];

Hv12 = Uad nc[number[d[]]-1, number[a[]]-1]
Hv13 = Uad nc[number[d[]]-1, number[b[]]-1]
Hv23 = Uad nc[number[b[]]-1, number[a[]]-1]

H = H0 + H1 + H2 + H3 + H12 + H13 + H23 + Hc1 + Hc2 + Hc3 + Hv12 + Hv13 + Hv23;

params = Join[params, {
 gammaPol1 -> Sqrt[(1/Pi) thetaCh[1] (extraGamma1) gammaA],
 gammaPol2 -> Sqrt[(1/Pi) thetaCh[1] (extraGamma2) gammaA],
 gammaPol3 -> Sqrt[(1/Pi) thetaCh[1] (extraGamma3) gammaA]
}];
```

Note that the model is defined with shifted onsite energies $\delta_i = \varepsilon_i + \frac{U_i}{2} + 2W_i$, such that half-filling corresponds to $\delta_i = 0$. Assuming a half-bandwidth of 1 eV (used as the energy unit), a typical parameter set used to calculate the

sub-gap spectrum and the expectation values is as follows:

```
[extra]
U1=0.20
U2=0.20
U3=0.20

delta1=0.1175
delta2=0.1175
delta3=0.1175

t12=0
Uad=0.05

Gamma1=0.02
Gamma2=0.02
Gamma3=0.02

[param]

bcsgap=0.0013

symtype=SPSU2

discretization=Z
z=1

Lambda=2
Tmin=1e-8

keepenergy=10
keep=4000
keepmin=4000

model=parallel_tqd.m

ops=n_d n_a n_b n_d^2 n_a^2 n_b^2 ...
```

The impurity spectral functions were calculated using the z-averaging with $N_z = 40$, `Tmin`=10^{-10} and the modified log-Gaussian kernel broadening (`smooth` = `newsc`), allowing for different broadening values within the superconducting gap ($\omega_0 = 3 \times 10^{-5}$) and above it ($\alpha = 0.05$).

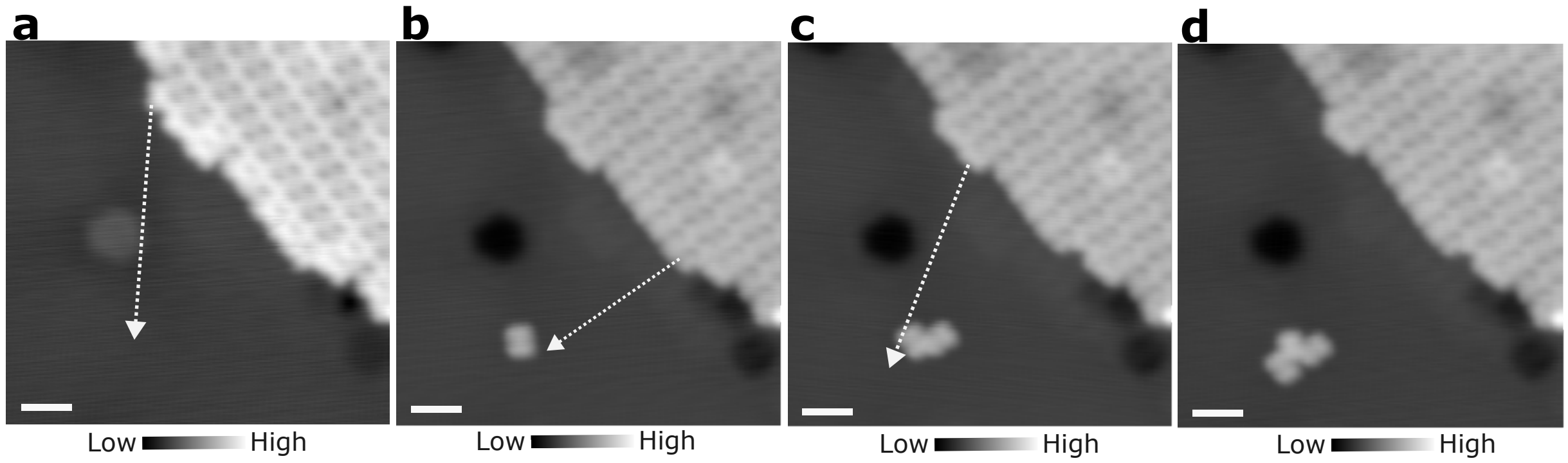

Supplementary Figure 2. | **Lateral manipulation of TBTAP molecules. a-d**, STM images showing the sequential construction of a trimer by laterally relocating individual TBTAP molecules from a molecular island. All images cover an area of 15×15 nm^2. The scale bar is 2 nm. Imaging parameters: **a,** $V_s = 1$ V, $I = 60$ pA; **b-d,** $V_s = 80$ mV, $I = 100$ pA.

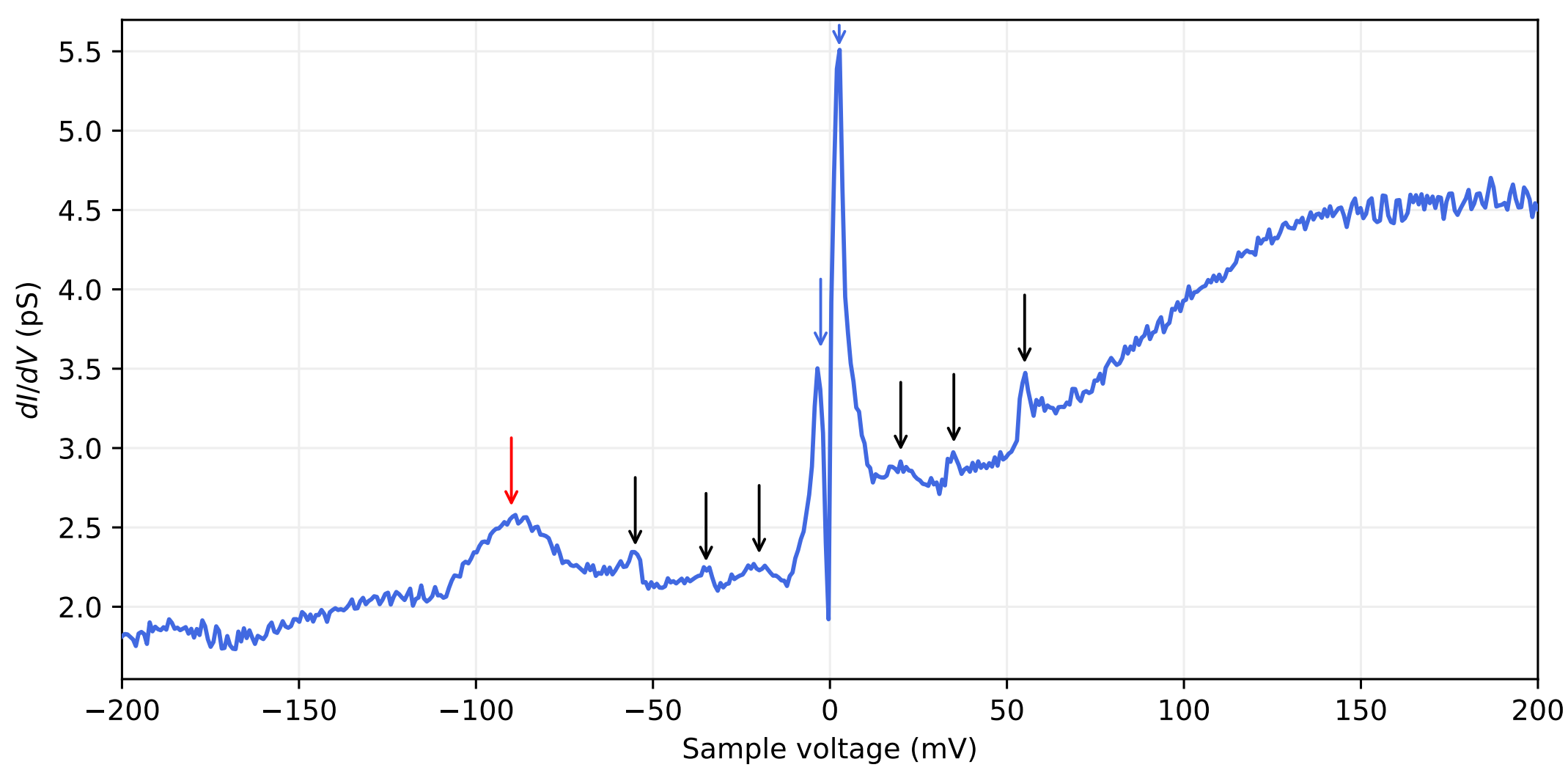

Supplementary Figure 3. | **Differential conductance spectra of a TBTAP trimer measured on -200 to 200 mV.** The dominant features at ± 2.7 mV (blue arrows) mark the coherence peaks at the edges of the superconducting gap. The conductance peaks at ± 20, ± 35 and ± 55 mV (black arrows) can be attributed to vibrations. The broader peak at -90 mV is attributable to the singly/lowest occupied molecular orbital (SOMO/LUMO). Note that as the data were measured using superconducting tip, all spectral features are shifted away from the Fermi energy energy by the tip gap $\Delta_t = 1.35$ meV. Spectra parameters: $V_s = 200$ mV, $A_{\rm mod} = 3$ mV and $f = 613$ Hz.

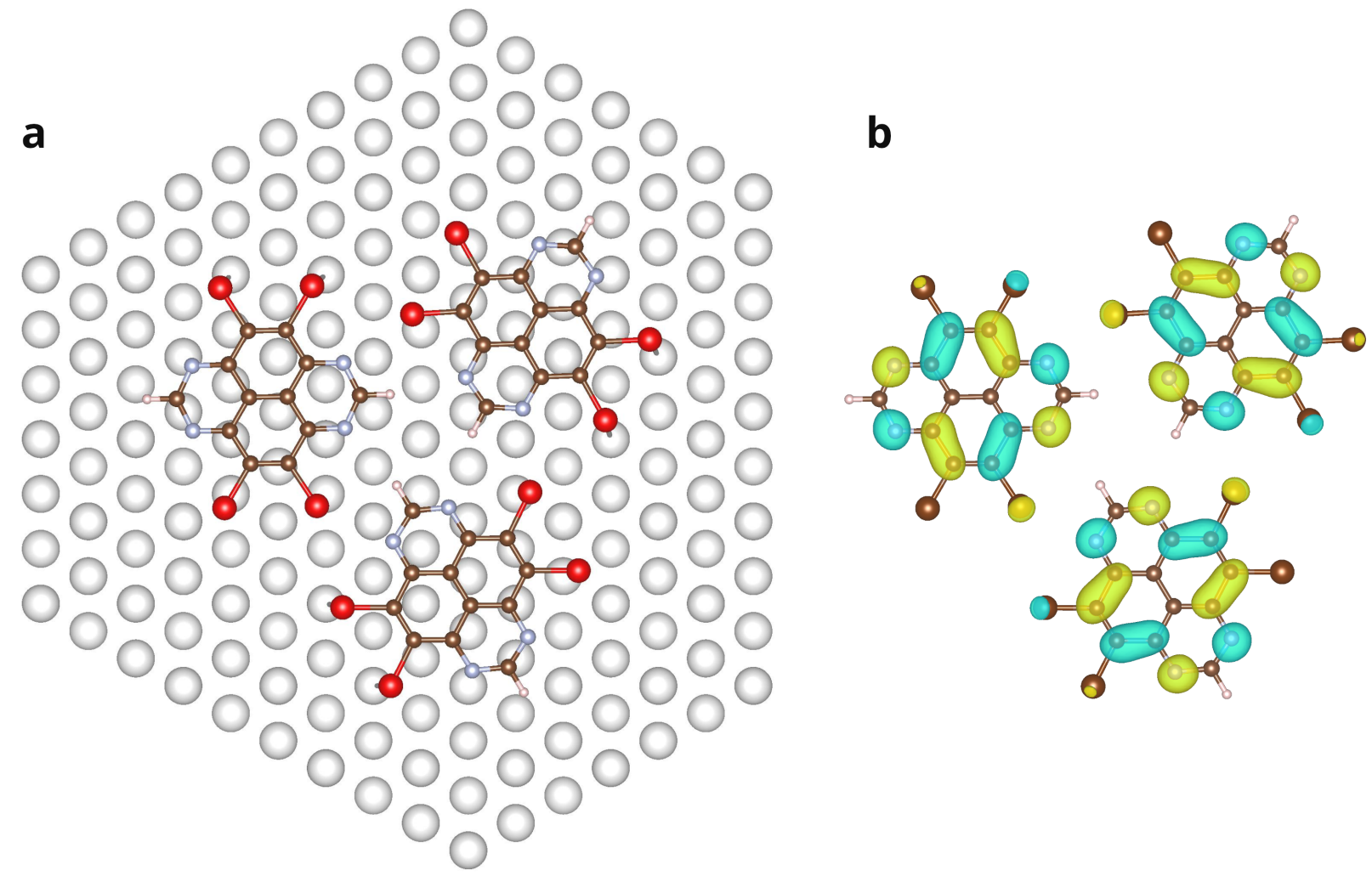

Supplementary Figure 4. | **DFT result on a TBTAP trimer. a**, Equilibrium position of a TBTAP trimer on Pb(111), that shows the chiral pattern as a result of the incommensurate size of the molecule with respect to the surface lattice parameters. Molecules form an equilateral triangle with 1 nm distance between the centers of mass of the molecules. The molecules' shorter axes are rotated by 16° with respect to the center of the triangle. Brown color represents carbon atoms, blue nitrogen, red bromine, pink hydrogen and white lead. **b**, Isosurface plot of the SOMO orbital (yellow - positive phase, turquoise - negative phase) of a TBTAP trimer charged by 3 electrons.

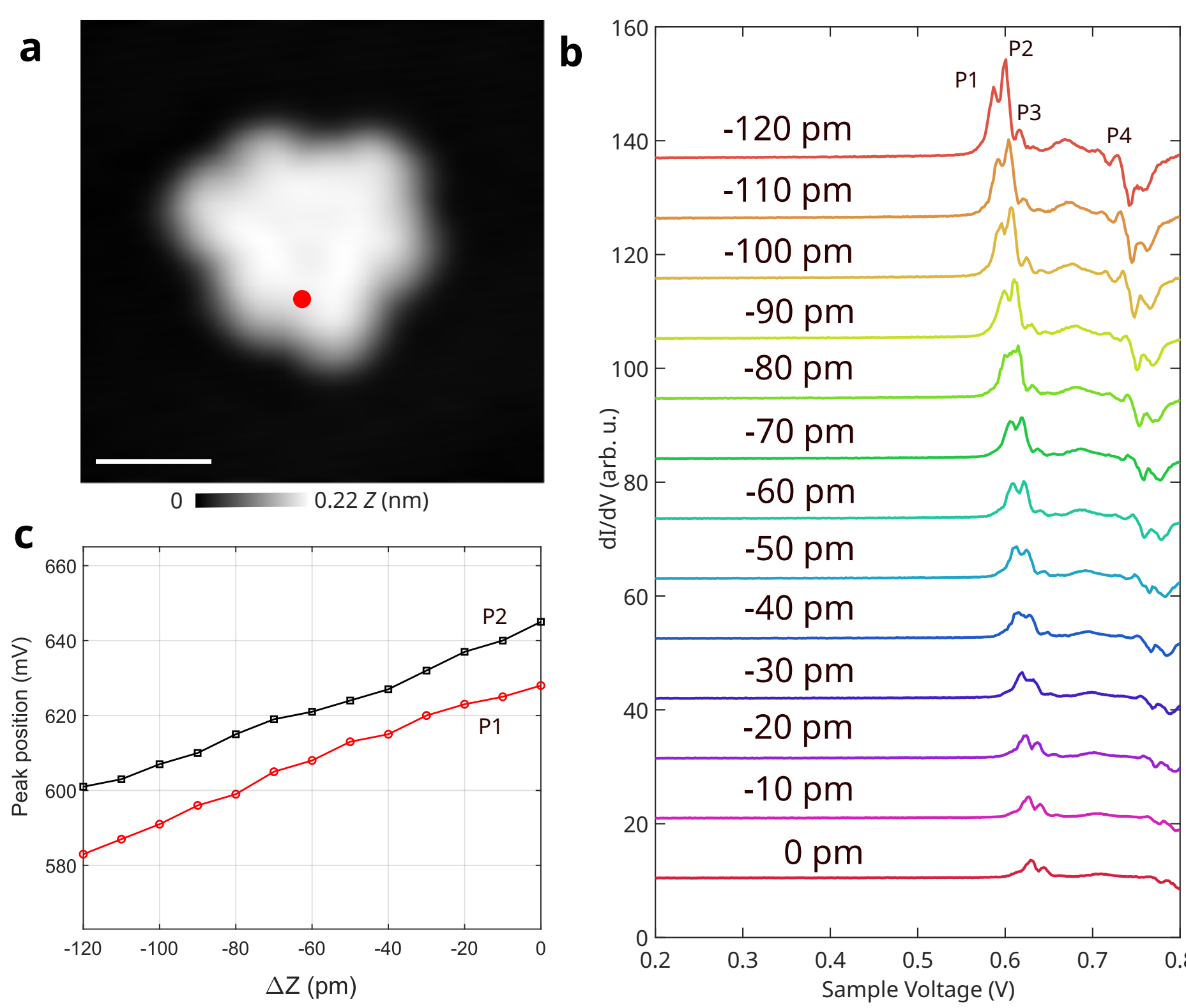

Supplementary Figure 5. | **Evolution of STS peaks during tip approach. a** and **b**, A set of high-energy STS spectra were acquired at the center of a molecule (indicated by the red dot) as the tip-molecule separation was gradually reduced. The spectra are vertically offset for clarity. Distinct spectral features are marked and labeled as P1 to P4. **c**, The variation in the energy positions of peaks P1 and P2 is plotted as a function of the tip approach distance. The STM image cover an area of 4×4 nm^2. The scale bar is 1 nm. Imaging parameters: $V_s = 100$ mV, $I = 100$ pA.

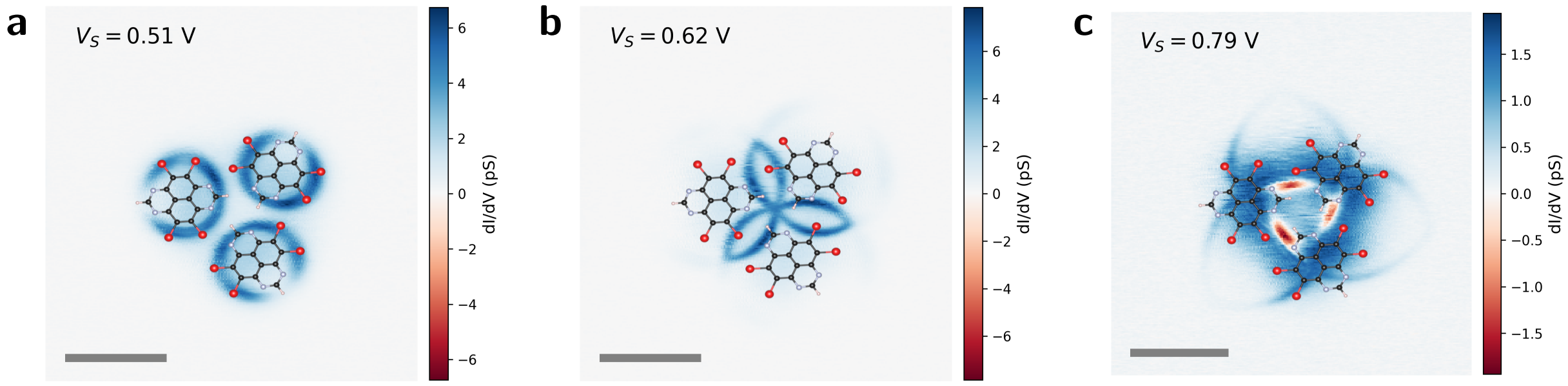

Supplementary Figure 6. | **Overlay of the molecular structure and discharging rings.** Approximate orientation of the molecules as obtained from DFT calculation with respect to the discharge rings for three values of the sample voltage $V_s = 0.51$ V (**a**), 0.62 V (**b**) and 0.79 V (**c**). The size of the molecules was slightly scaled along the vertical axis to fit the charging rings. This small discrepancy is probably a result of an imperfect calibration of the experimental setup. The scale bar is 1 nm.

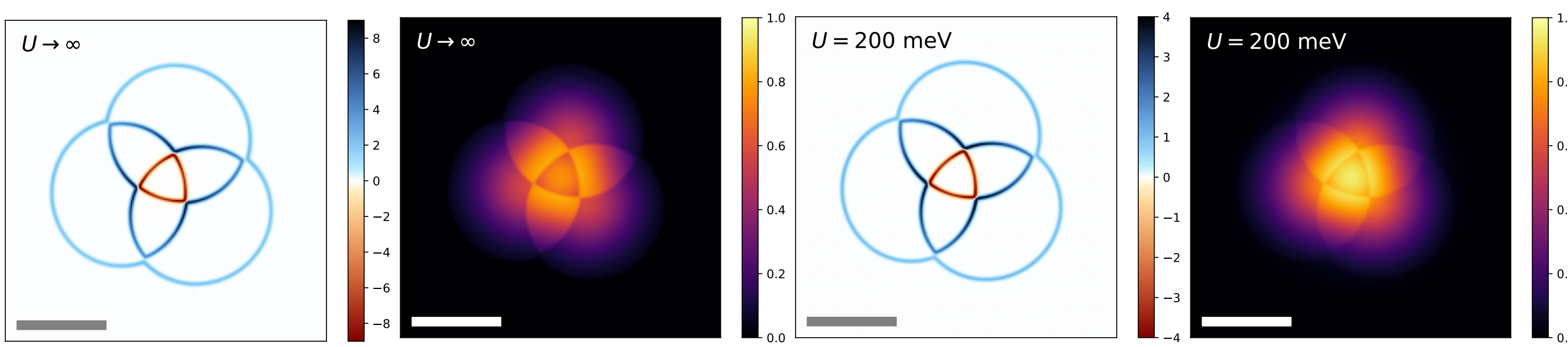

Supplementary Figure 7. | **Effect of finite charging energy.** Comparison of the Pauli ME results on $\mathrm{d}I/\mathrm{d}V$ and current (both in arb. units) for $V_s = 0.72$ V for infinite value of the on-site Coulomb interaction U, which prohibits double occupancy of a site, with result for $U = 200$ meV. The finite value of the on-site Coulomb interaction leads to larger values of the current, but to negligible differences in $\mathrm{d}I/\mathrm{d}V$. Data were calculated using the `QmeQ` package [11]. The scale bar is 1 nm.

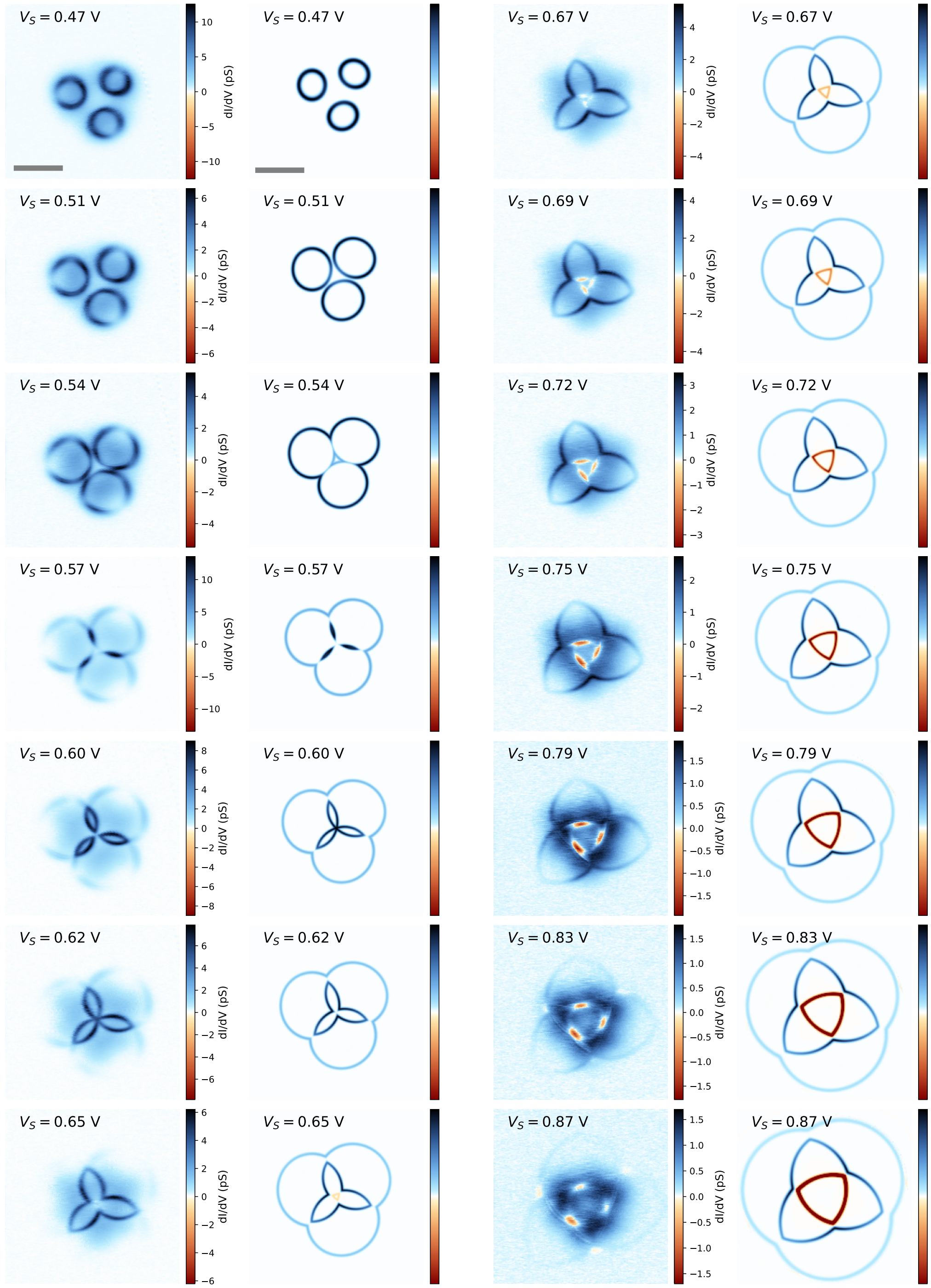

Supplementary Figure 8. | **Spatial variation of differential conductance in a TBTAP trimer at varying bias voltage - experiment vs. theory.** First and third column: Experimental $\mathrm{d}I/\mathrm{d}V$ maps of a TBTAP trimer at different sample voltage V_s. The red color represents regions of NDC. Note the different maxima on the color scales. Second and fourth column: Simulation of the charging rings using the PME approach (in arb. u.). Here, the effects of the TBTAP orbital structure are omitted for clarity. The scale bar is 1 nm.

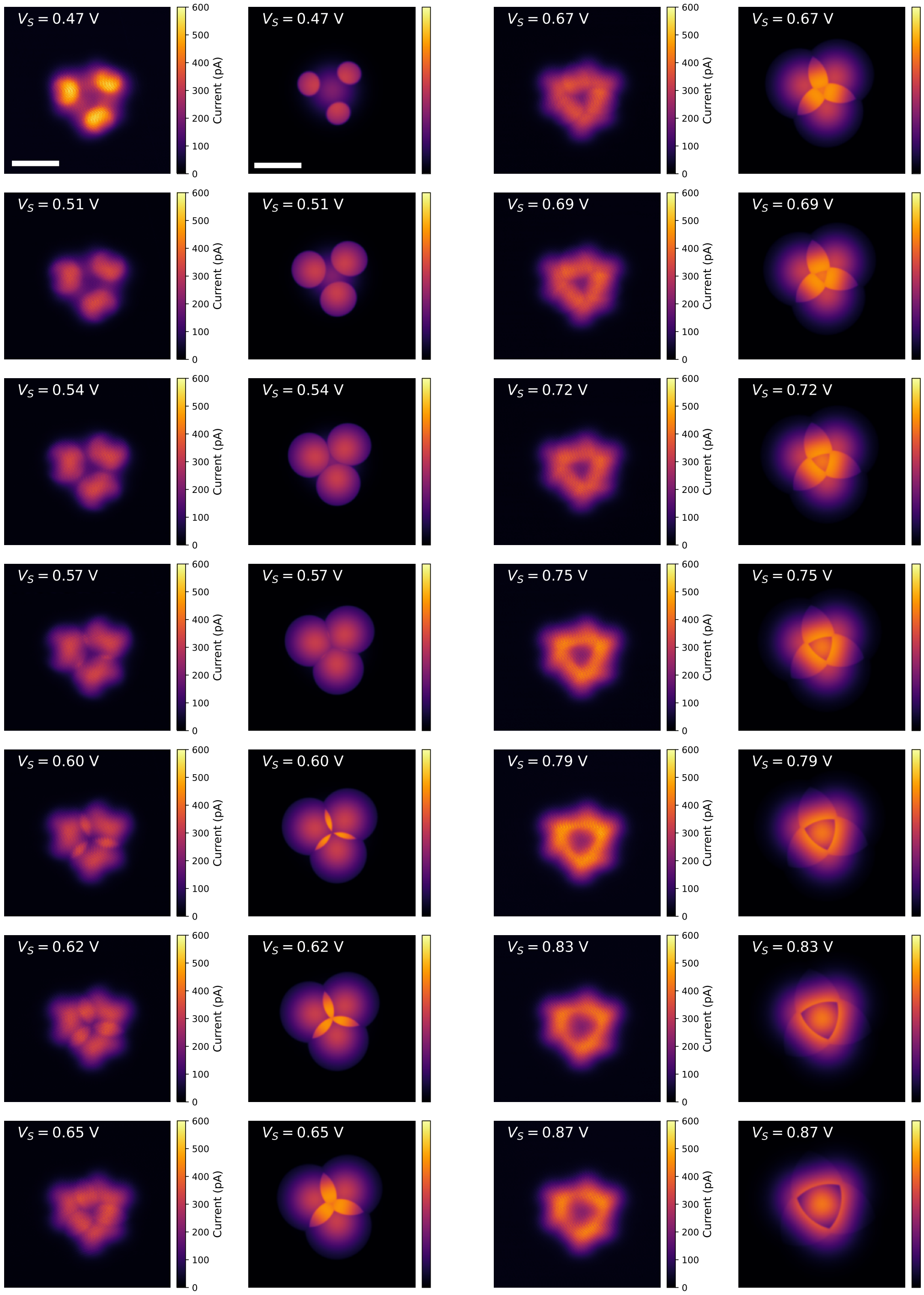

Supplementary Figure 9. | **Spatial variation of STM current in a TBTAP trimer at varying bias voltage - experiment vs. theory.** First and third column: STM current maps from experiment on a TBTAP trimer at the same sample voltages V_s as in Supplementary Fig. 8. Second and fourth column: STM current maps simulated using PME approach (in arb. u.). The color scales have the same maxima for easier comparison. The scale bar is 1 nm.

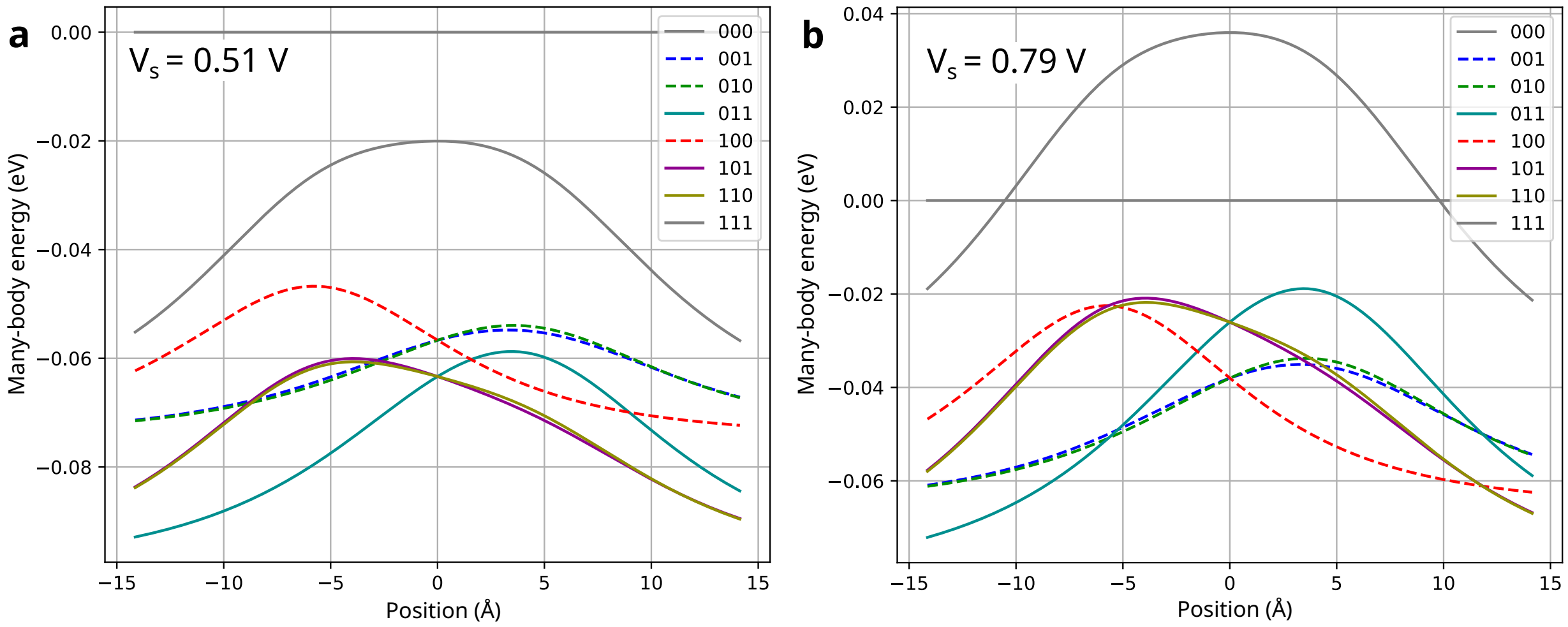

Supplementary Figure 10. | **Many-body energies of the isolated trimer.** Many-body spectra calculated for sample voltages $V_\mathrm{s} = 0.51$ V (**a**) and 0.79 V (**b**) along the same path as in Figs. 3a-d in the main text, that include the effects of the quadrupole moment. As a result, the singly and doubly occupied states are slightly split, in contrast to the data shown in Fig 3a. Results in panel **a** also show that the ground state for lower voltages is always doubly occupied, as noted in Fig. 3d in the main text. The triply occupied state lies higher in energy due to the sizable inter-site Coulomb coupling.

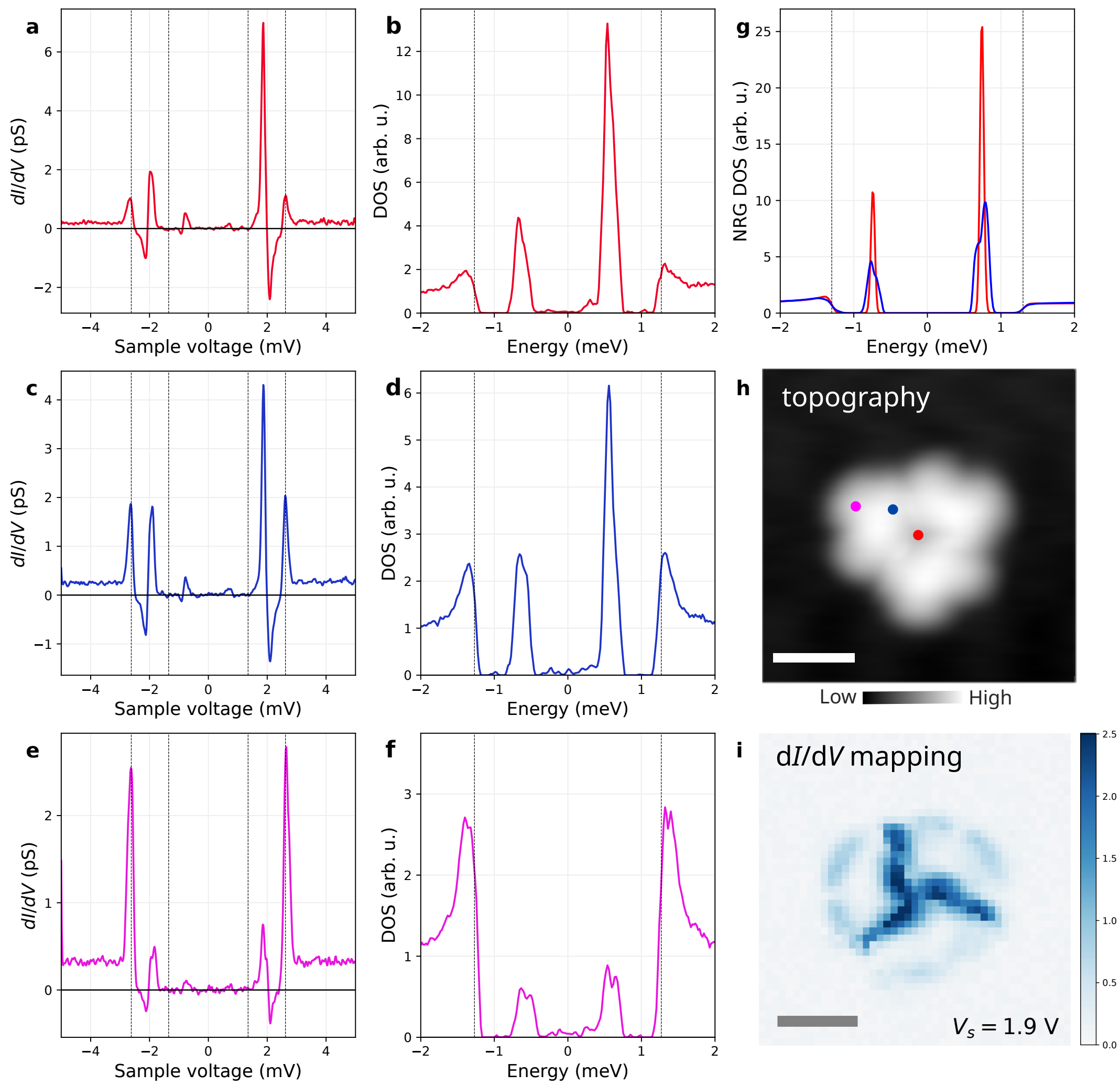

Supplementary Figure 11. | **YSR spectra of TBTAP trimers. a-f**, $\mathrm{d}I/\mathrm{d}V$ spectra measured at the positions marked by red, blue, and violet dots in panel **h** and the corresponding surface density of states (DOS). In addition to the coherence peaks at sample voltages $V_\mathrm{s} = \pm 2.7$ mV, two pairs of YSR states appear at $V_\mathrm{s} = \pm 1.85$ mV and $V_\mathrm{s} = \pm 1.95$ mV. Faint features observed at $V_\mathrm{s} = \pm 0.75$ mV are thermal replicas of the YSR peaks. Vertical lines in panel **a** mark the values of $\pm\Delta_\mathrm{t}$ and $\pm(\Delta_\mathrm{s} + \Delta_\mathrm{t})$. Vertical lines in panel **b** mark the gap edges $\pm\Delta_\mathrm{s}$. **g**, NRG result on the YSR spectra of the superconducting TIAM. Model parameters read $\Delta = 1.3$ meV, $U = 200$ meV, $W = 50$ meV, $\varepsilon = -104$ meV, $t = 0$ and $\Gamma_2 = \Gamma_3 = 20$ meV. Red line: symmetric case ($\Gamma_1 = 20$ meV). Blue line: slightly asymmetric case ($\Gamma_1 = 19.5$ meV) to simulate the effect of the scanning tip. The asymmetry leads to the splitting of the YSR peaks. **h**, Topographic image of the TBTAP trimer. **i**, Spatially resolved $\mathrm{d}I/\mathrm{d}V$ map acquired at $V_\mathrm{s} = 1.9$ mV, illustrating the chiral nature of the YSR state. The scale bars are 1 nm.

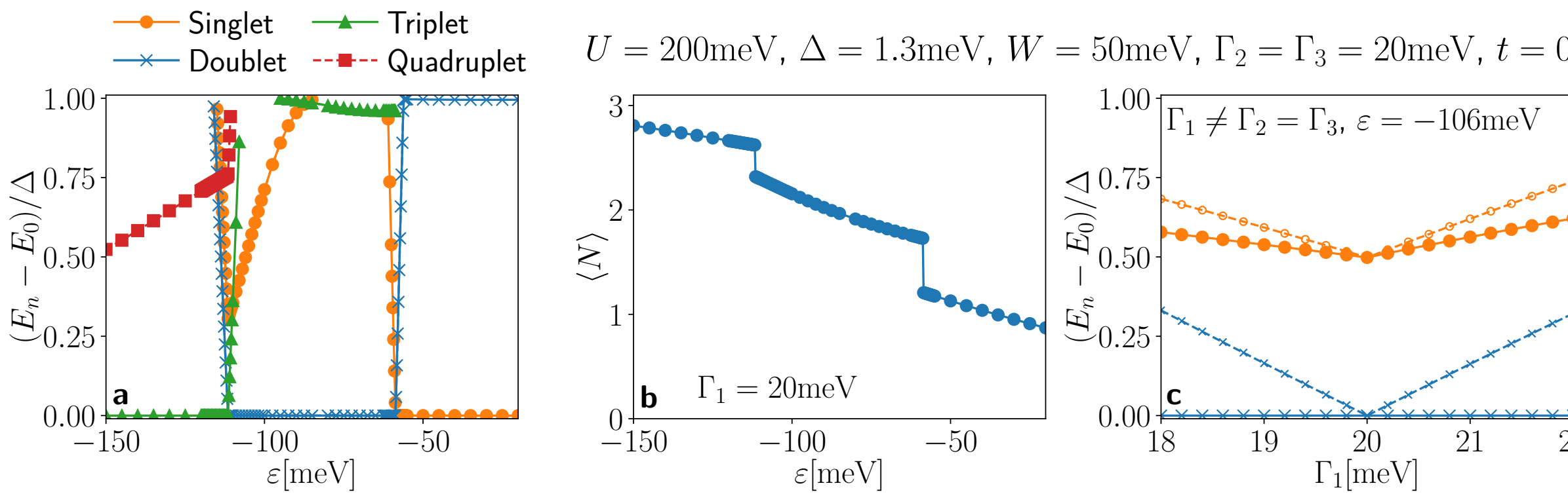

Supplementary Figure 12. | **Evolution of YSR states in TBTAP trimers.** **a**, Many-body state energies as functions of the local energy ε measured with respect to the ground state. The color encodes the state degeneracy: singlet (orange), doublet (blue), triplet (green) and quadruplet (red). The quantum phase transitions at $\varepsilon = -58.5$ meV and $\varepsilon = -111.5$ meV are marked by the change of the ground state degeneracy. Model parameters read $U = 200$ meV, $W = 50$ meV, $\Gamma_1 = \Gamma_2 = \Gamma_3 = 20$ meV and $t = 0$. **b**, Total charge of the trimer as function of the local energy ε. The total charge for $\varepsilon = -90$ meV is two, in agreement with the PME result. **c**, The effect of asymmetry induced by varying coupling to the substrate Γ_1 of one of the molecules for $\varepsilon = -106$ meV. Such variation can be induced by the presence of a scanning tip and leads to a splitting of the YSR energies, in agreement with the experimental result. The line colors follow panel a (orange - singlet, blue - doublet).

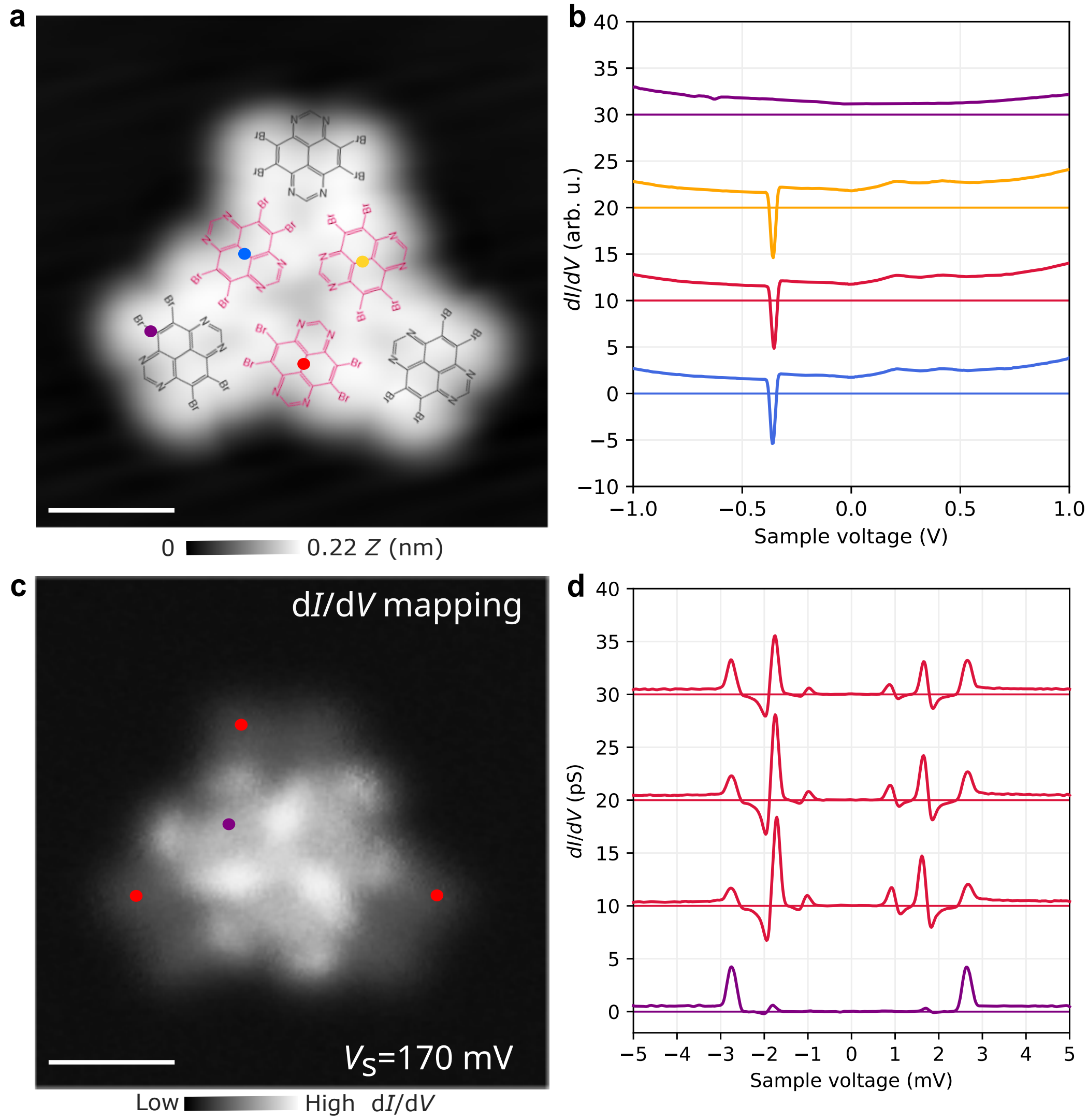

Supplementary Figure 13. | **Structure of a TBTAP hexamer.** **a**, STM image and structural model of a triangular hexamer constructed from six TBTAP molecules. (I = 100 pA, V_s = −600 mV) **b**, dI/dV spectra measured on three inner and one outer TBTAP molecules at the positions marked by dots in panel a. Pronounced charging dips are observed between −400 mV and −500 mV for the inner molecules, while no significant features appear for the outer molecule. In addition to the dips at negative V_s, two resonances are observed at V_s = 170 mV and V_s = 330 mV, which can be attributed to the LUMO and LUMO+1 energy levels of the inner molecules of the hexamer. The scale bar is 1 nm. **c**, Constant-height dI/dV map acquired at V_s = 170 mV, highlighting the spatial variation in electronic structure between the inner and outer TBTAP molecules. **d**, YSR spectra measured on one inner and three outer TBTAP molecules at positions marked by dots in panel **c**. The inner molecule shows no YSR peaks, revealing the loss of its radical nature.

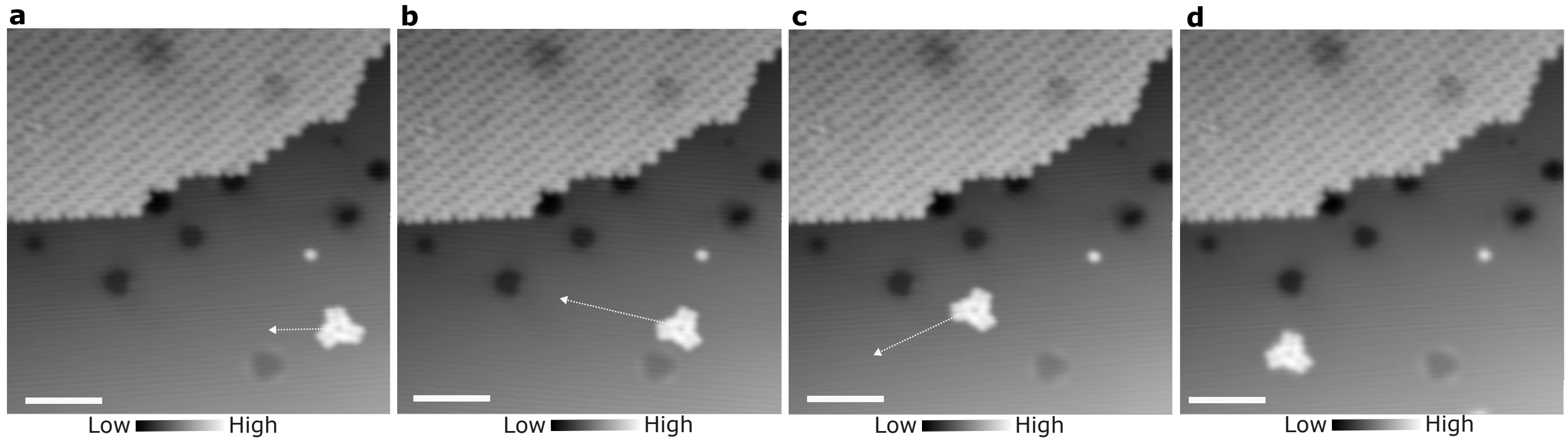

Supplementary Figure 14. | **Movement of the hexamer assembly using lateral manipulation. a-d**, STM images showing the sequential lateral movement of a hexamer structure, composed of six TBTAP molecules, demonstrating its structural integrity. The hexamer is stabilized through a combination of hydrogen and halogen bonding. Images cover an area of 25×25 nm^2. The scale bar is 5 nm. Imaging parameters: (**a**) $V_\mathrm{s} = 60$ mV, $I = 80$ pA; (**b-d**) $V_\mathrm{s} = 80$ mV, $I = 100$ pA.

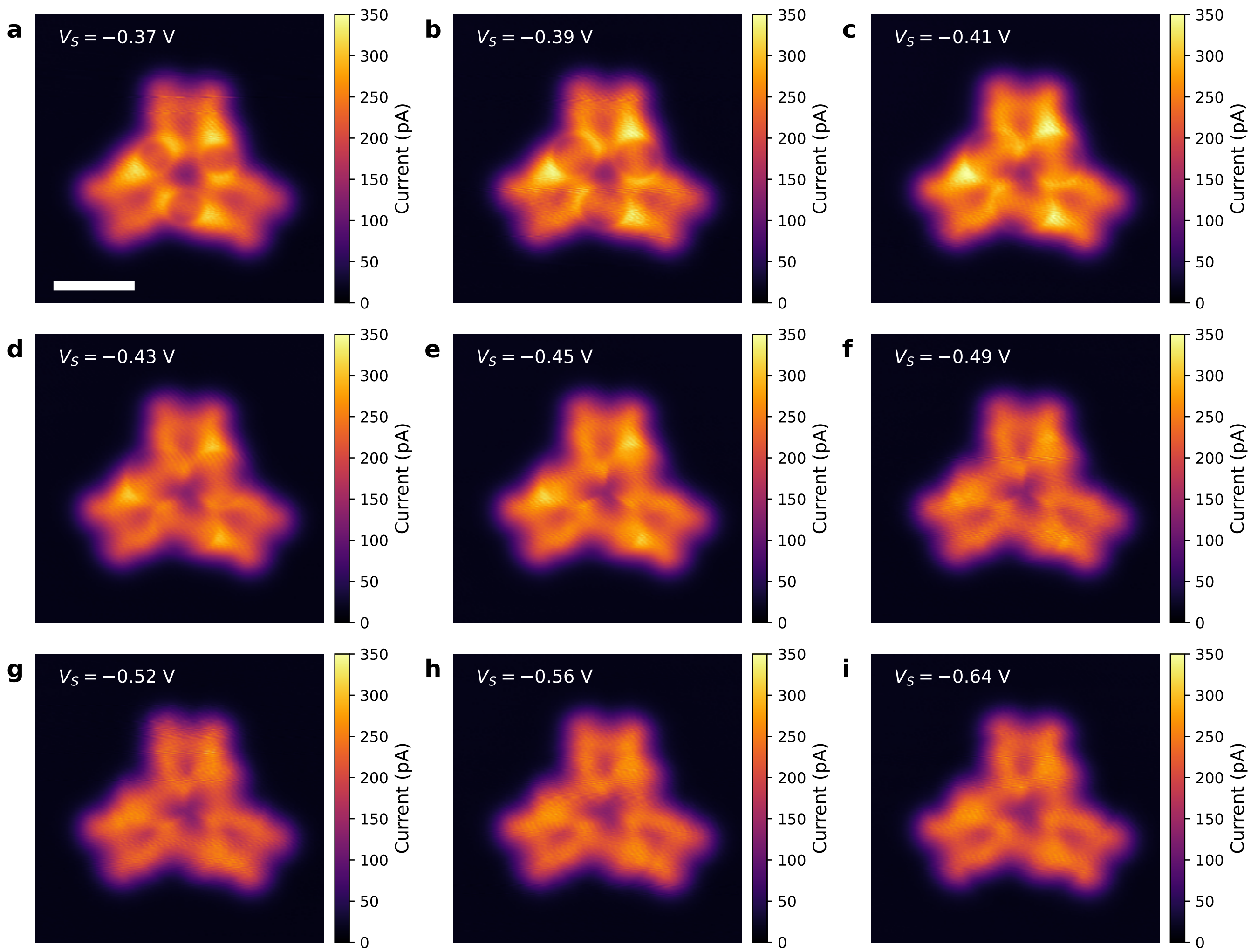

Supplementary Figure 15. | **Spatial variation of current in a TBTAP hexamer at varying bias voltage. a-i**, Spatial maps of the absolute value of the current plotted for the same parameters as in Fig. 4 in the main text. The chiral structure of the hexamer is more pronounced than in the dI/dV maps. The color scales have the same maxima for easier comparison. The scale bar is 1 nm.

[1] C. Argento and R. H. French, Parametric tip model and force–distance relation for Hamaker constant determination from atomic force microscopy, J. Appl. Phys. **80**, 6081–6090 (1996).

[2] R. Baier, C. Leendertz, M. C. Lux-Steiner, and S. Sadewasser, Toward quantitative Kelvin probe force microscopy of nanoscale potential distributions, Phys. Rev. B **85** (2012).

[3] D. Z. Gao, J. Grenz, M. B. Watkins, F. Federici Canova, A. Schwarz, R. Wiesendanger, and A. L. Shluger, Using metallic noncontact atomic force microscope tips for imaging insulators and polar molecules: Tip characterization and imaging mechanisms, ACS Nano **8**, 5339–5351 (2014).

[4] L. Gross, B. Schuler, F. Mohn, N. Moll, N. Pavliček, W. Steurer, I. Scivetti, K. Kotsis, M. Persson, and G. Meyer, Investigating atomic contrast in atomic force microscopy and Kelvin probe force microscopy on ionic systems using functionalized tips, Phys. Rev. B **90** (2014).

[5] O. Krejčí, P. Hapala, M. Ondráček, and P. Jelínek, Principles and simulations of high-resolution STM imaging with a flexible tip apex, Phys. Rev. B **95** (2017).

[6] C. J. Chen, Tunneling matrix elements in three-dimensional space: The derivative rule and the sum rule, Phys. Rev. B **42**, 8841–8857 (1990).

[7] J. Doležal, S. Canola, P. Hapala, R. C. de Campos Ferreira, P. Merino, and M. Švec, Real space visualization of entangled excitonic states in charged molecular assemblies, ACS Nano **16**, 1082–1088 (2021).

[8] N. Oinonen, A. V. Yakutovich, A. Gallardo, M. Ondráček, P. Hapala, and O. Krejčí, Advancing scanning probe microscopy simulations: A decade of development in probe-particle models, Comput. Phys. Commun. **305**, 109341 (2024).

[9] B. Goldozian, F. A. Damtie, G. Kiršanskas, and A. Wacker, Transport in serial spinful multiple-dot systems: The role of electron-electron interactions and coherences, Sci. Rep. **6**, 22761 (2016).

[10] C. Li, V. Pokorný, M. Žonda, J.-C. Liu, P. Zhou, O. Chahib, T. Glatzel, R. Häner, S. Decurtins, S.-X. Liu, R. Pawlak, and E. Meyer, Individual assembly of radical molecules on superconductors: Demonstrating quantum spin behavior and bistable charge rearrangement, ACS Nano **19**, 3403 (2025).

[11] G. Kiršanskas, J. N. Pedersen, O. Karlström, M. Leijnse, and A. Wacker, QmeQ 1.0: An open-source Python package for calculations of transport through quantum dot devices, Comput. Phys. Commun. **221**, 317 (2017).

[12] R. Žitko, NRG Ljubljana (2021).
