# Supplementary material for: Non-equilibrium correlated electron dynamics in triangular molecular assemblies
Source: Nat Commun. 2026 Jul 8;17:8406. doi: 10.1038/s41467-026-75051-3 (PMC13473136; doi:10.1038/s41467-026-75051-3)
Supplement: Supplementary file 2 [file 41467_2026_75051_MOESM2_ESM.pdf]

nature portfolio

Peer Review File

Non-Equilibrium Correlated Electron Dynamics in Triangular Molecular Assemblies

Corresponding Author: Dr Vladislav Pokorný

This file contains all reviewer reports in order by version, followed by all author rebuttals in order by version.

Version 0:

Reviewer comments:

Reviewer #1

(Remarks to the Author)
Charging rings in surface-adsorbed molecular systems present intriguing properties that have been tackled in many publications. However, still a lot of details in the experimental data remain to be understood. This paper is a highlight example of a careful investigation of these so-called charging rings. The authors observe both an increase or decrease of the tunneling current along the ring depending on lateral position of the STM tip along a three-molecule configuration. Supported by calculations, they show that this behavior originates from interactions among the molecules, leading to charge rearrangements and population of many-body states that can suppress the tunneling current. The model structure of three molecules is ideal in that it is very well defined and calculations can capture this system. However, the theoretical foundations are applicable to extended structures. I recommend publication of this paper in Nature Communications, but would like to request some more detailed descriptions as commented below:
The colors in the background of figure 3a seem to suggest the ground state of the molecular cluster depending on tip position. If this is correct, I wonder why the background in section 3 with one electron on the cluster has three distinct areas (010, 010, 100) rather than the 010 and 001 being at the same energy? Or am I missing something? Similar questions apply to region 2 and 4. In any case, a more detailed explanation of the areas would be helpful.
The explanation of the contribution to the tunneling current in figure 3b is not entirely clear to me. Are the colored contributions relative contributions or some absolute values? The current seems to vary already along the red molecule. Why? Where does the exponential dependence enter or where can one see it in the plot? The description of figures and their meaning is minimal in the paper. More details should be presented in the revised version.
Very early on in the manuscript, the authors assume the ring to be a discharging process. They may want to stress that the molecules are charged in their ground state as can be deduced from the YSR spectra already at this point, possibly even including the spectra in the main manuscript. They may also want to comment on the origin of the splitting of the YSR states.

(Remarks on code availability)

Reviewer #2

(Remarks to the Author)
The authors study clusters of TBTAP molecules deposited on a superconducting substrate Pb(111) surface, and laterally manipulated using an STM tip. The molecules are arranged with atomic precision into an equilateral triangle configuration, with 1nm distance separating molecule centres. Low-temperature scanning tunnelling spectroscopy is used to create a real-space map of the tunnelling current, which provides insights into charging/discharging effects of the molecular cluster, due to the electric field of the STM tip and applied voltages. A complex structure of charging rings is uncovered, including chiral features and regions of negative differential conductance. In particular, charging rings are shown to arise from internal charge redistribution within the cluster without changes to the total charge.

The authors perform careful and sophisticated modelling of these effects, taking into account quantum many-body effects beyond the often-employed single-particle picture. They model the triangular molecular cluster as a three-impurity Anderson model, including both on-site Coulomb interaction U and capacitive inter-site repulsion W, as well as molecule-substrate hybridization and coupling to the STM tip, and modulations to the local electric field coming from the STM tip. This is an

impressive level of theory -- absolutely essential to capture many-body effects and Coulomb blockade as well as experimentally observed transport features at low temperatures. In equilibrium, NRG is used to characterize the system, which fully takes into account interaction-renormalization effects and molecule-substrate entanglement. This treatment goes beyond weak coupling approximations and captures non-Markovian effects. However, it is restricted to equilibrium. To capture finite-bias transport and non-equilibrium occupation effects, the authors also employ the PME method, which is Markovian and valid only at weak coupling, but does capture essential features of the experiment in the appropriate limit. It should be noted that this is already state-of-the-art and no computational method exists to treat strong correlations, strong substrate coupling, and finite bias exactly and on an equal footing. Therefore I find this treatment impressive. The good match between theory and experiment also validate and justify the approach and methodologies used, which I believe are entirely appropriate.

The key findings are that many-body collective effects due to strong electron correlations, produce complex STS signatures and charging effects that cannot be described within a single-particle picture. This is an impressive work, with interesting results, and an excellent marriage of theory and experiment. The manuscript itself is also well-written. This work is entirely suited for publication in Nature Communications (or Nature Physics for that matter). I have the following questions and suggestions to improve the manuscript and analysis:

1) In terms of the context of the present work, it would help to mention highly relevant related work on quantum dot trimers (triple quantum dots). Although the physical system and experimental measurement is different, the underlying theoretical model is basically the same. In particular, triangular TQDs are well-known to have multiple collective states (for example overall spin doublet states with different chirality but the same electron filling) which are induced by strong correlations. Importantly, the ground state can switch between these, and this shows up in transport. See e.g. PRB 80, 155330 (2009); PRB 79, 085124 (2009); PRB 81, 075126 (2010).

2) As the authors point out, transport in multi-orbital systems (molecular or QD) is often discussed in terms of simplistic single-particle quantum interference. However, for systems which show Coulomb blockade or Kondo due to strong electron correlations, this is totally insufficient to explain basic charging or transport effects. This is an important point, but it should be pointed out that quantum interference effects are also important for interacting systems -- but one must now think about many-body quantum interference in Fock space. The proper transport problem is not simply about rearranging charges in many-body states, but about complex transition amplitudes between Dyson orbitals. The present findings fit in nicely with these theories, but I suggest placing the current work in the proper context. See e.g. PRB 90, 125413 (2014); Nature Communications 8, 15210 (2017); PRL 133, 076501 (2024).

3) Each TBTAP molecule is treated as just a single quantum orbital (Anderson impurity). Obviously a full ab initio treatment of the entire molecule (or cluster) would be far out of reach when taking into account interactions, but how/why is a single-orbital approximation appropriate? What about the real-space structure of the molecular orbital in question? The approximation appears to be justified post hoc by comparison with experiment, but what is the physical reason for this drastic simplification?

4) Why is a superconducting substrate used, and what role does it play here? It would seem to me that this could complicate transport signatures significantly, relative to a standard metallic single-molecule transistor setup.

5) For the NRG studies, more details of the implementation and calculations performed must be provided in the Methods or Supplementary Information section. All model and NRG parameters as well as further corroborating data should be provided. For the theory section in the Supplementary Information, what is the justification for the transport formulae used? Why not use Landauer-Buttiker or Meir-Wingreen for a more rigorous description? In the section detailing the model, more discussion should be provided on why this form of the hybridization matrix is used. Given the off-diagonal substrate hybridization terms, do we end up with a complicated multi-channel Kondo model? Or is it tacitly assumed that all three molecules couple to the same bath orbital, making it a single-channel problem (plus the STM tip). If the latter, what is the justification for this? Why are there no off-diagonal hybridization terms with the tip?

6) For the PME calculations, how is the weak-coupling approximation justified and why does it work so well? In particular, the temperature is rather low, and so system-substrate entanglement and renormalization effects would be generically expected (these are the effects captured by NRG). The physics should be non-Markovian at low T, even for weak coupling. Indeed, we are told that Gamma>10T and so this is really not the sequential tunnelling regime. How is Gamma quantified here and what is its physical meaning? One explanation could be that if by Gamma you are taking the standard quantum impurity definition Gamma=\pi\rho_0 V^2 where V is the tunnel-coupling between system and bath and \rho_0 is the bath density of states at the Fermi level… then rho_0 is strongly suppressed by the superconducting gap of the substrate and tip. Thus one suppresses Kondo and other strong-coupling effects and weak-coupling methods are remarkably accurate, see e.g. PRL 107, 256802 (2011).

7) It is stated that the “full” Hilbert space of the molecular cluster is 4^3=64 states but that the large Coulomb interaction suppresses double occupancy and reduces this to 2^3=8 which is tractable. I would make a few comments here. First, it should be made clear that the actual Fock space dimension of each molecule is enormous and far beyond reach of exact diagonalization or ab initio methods; the local Fock space dimension of 4 is already a vast simplification by keeping just the SOMO (and this will be an approximation since there will be interaction effect between all Mos). Secondly, 4^3=64 is still a tiny Fock space in the context of many-body problems, and is fully tractable. The issue is, rather, that coupling to the substrate and STM tip beyond weak-coupling (Markovian) approximation leads to genuine non-perturbative many-body effects because now one has to deal with the lead electrons as well. This is exactly the physics taken into account exactly by

NRG. Finally, I do not really understand the reduction to 2^3=8 states. Precluding double occupancy leaves 3 states on each SOMO so I would have thought this is 3^3=27 states remaining that need to be described (up, down, empty).

8) The mechanism for the tip-induced charge reorganization on the trimer is interesting and obviously a crucial for the story. In the N=2e sector, the interpretation presented by the authors is that the tip potential pushes out the electron on the site below it, onto the remaining two molecules. However, if there is no direct tunnelling and we have effectively 3 degenerate, localized 2-electron states |1,1,0>, |1,0,1>, |0,1,1> then this charge reorganization (which I am happy to accept does happen, given the experimental evidence!) must happen by tunnelling into and then out of the substrate. Is that so? My issue here is that the substrate is superconducting and therefore at low T can only accept or release cooper pairs. Single-electron tunnelling is suppressed inside the SC gap Delta. So the charge reconfiguration is maybe happening by tunnelling a *pair* into the substrate, and then back out into different molecule states? Another possibility is that the actual quantum N=2e state is a superposition of |1,1,0>, |1,0,1>, |0,1,1> from the start, and so the tip field is just re-adjusting the relative weights of these rather than causing explicit tunnelling events. The situation is even more confusing for N=1e, because here there is no pair that can tunnel directly. Here is seems more likely that the N=1e state is actually a quantum superposition of “classical” states with 1e on either one of the molecules. This should be addressed and discussed in the manuscript and supporting evidence for the mechanism provided where possible.

(Remarks on code availability)
--

Reviewer #3

(Remarks to the Author)
In this manuscript, Li et al report a work of using STM tip to spatially map out characteristic peaks and dips in differential conductance (dI/dV) on trimer clusters of TBTAP molecules on a Pb(111) substrate. Using highly complicated theoretical modelling, they provide an explanation of the experimental observation. They argue, instead of the common mechanism of charging and discharging, the dI/dV peak/dip are attributed to charge rearrangement among the three molecules in the cluster. They conclude that this work “shed new light on fundamental quantum many-body effects in strongly interacting molecular systems and open pathways for engineering novel electronic phases by controlling cluster topology and the electrostatic environment.”
The experimental data presented in the manuscript are of very high quality. However, the theoretical arguments are very complex and difficult to follow for the reviewer. The following comments are mostly about the theoretical arguments:
1. On Page 2: the reviewer finds “we directly visualize discharging processes and characteristic Coulomb ring patterns associated with single-electron charging events, including the onset of robust negative differential conductance (NDC) near the center of the trimer.” And “the (dis)charging rings and conductance discontinuities do not correspond to a change in the total charge of the trimer. Instead, they originate from subtle intra-cluster charge rearrangements, where electrons are redistributed among the molecules without altering the net charge.” So, it seems that the trimer does not gain or loss charge in the whole process. If this is the case, the author shall not use charging/discharging, which confuses the readers.
2. On Page 4, the reviewer reads “charging the cluster with two electrons to −2e more energetically favorable than charging it with three electrons to −3e”. and “Upon applying a sufficiently high bias voltage (sample voltage Vs = 0.6 V when tip is at the center of the cluster), an additional electron is repelled from the cluster, leading to a net charge of −1e, meaning that only one site remains occupied.” This is the key point. Experimentally, these charge states can be confirmed using Kelvin parabolas showing the LCPD using nc-AFM. To unambiguously verify the proposed mechanism, such measurements are essential.
3. The total charge of the cluster for different regions is marked in Figure 3d, showing at the two sides, the charge is 2. However, Figure 3b shows at the left side the charge is 2 as labelled with “011”, while at the right side the charge is 1 as labelled with “001”. The labels are confusing. Also, Fig. 3b shows 011->111, but this is not discussed in the text.
4. The discussion of Fig. 3a is very brief and general, only five lines on page 4. Based on these five lines, the reviewer does not understand the very complex features of Fig. 3a. Similar situation is encountered for Fig. 3b.
5. It is not clear for the reviewer how the bias voltage of the STM junction is treated in the modelling. Considering the major arguments heavily rely on the modelling, a concise presentation of the Hamiltonian and/or schematic configuration of the system shall be presented in the main text.

(Remarks on code availability)

Version 1:

Reviewer comments:

Reviewer #1

(Remarks to the Author)
The authors have clarified all my questions. I recommend publication of the paper as is.

(Remarks on code availability)

Reviewer #2

(Remarks to the Author)
I thank the Authors for their detailed replies to my questions and comments. I am satisfied by these and believe the revisions to the manuscript have improved the work.
I have also reviewed the replies to the questions from the other referees and find these convincing also. Therefore I recommend publication in present form.

(Remarks on code availability)

Reviewer #3

(Remarks to the Author)
My previous comments are satisfactorily addressed in the revision. I support to accept this manuscript for publication in Nature Commun.

(Remarks on code availability)

Open Access This Peer Review File is licensed under a Creative Commons Attribution 4.0 International License, which permits use, sharing, adaptation, distribution and reproduction in any medium or format, as long as you give appropriate credit to the original author(s) and the source, provide a link to the Creative Commons license, and indicate if changes were made.
In cases where reviewers are anonymous, credit should be given to 'Anonymous Referee' and the source.
The images or other third party material in this Peer Review File are included in the article's Creative Commons license, unless indicated otherwise in a credit line to the material. If material is not included in the article's Creative Commons license and your intended use is not permitted by statutory regulation or exceeds the permitted use, you will need to obtain permission directly from the copyright holder.
To view a copy of this license, visit https://creativecommons.org/licenses/by/4.0/

Reply to referees: Non-Equilibrium Correlated Electron Dynamics in Triangular Molecular Assemblies

Referee 1

Charging rings in surface-adsorbed molecular systems present intriguing properties that have been tackled in many publications. However, still a lot of details in the experimental data remain to be understood. This paper is a highlight example of a careful investigation of these so-called charging rings. The authors observe both an increase or decrease of the tunneling current along the ring depending on lateral position of the STM tip along a three-molecule configuration. Supported by calculations, they show that this behavior originates from interactions among the molecules, leading to charge rearrangements and population of many-body states that can suppress the tunneling current. The model structure of three molecules is ideal in that it is very well defined and calculations can capture this system. However, the theoretical foundations are applicable to extended structures. I recommend publication of this paper in Nature Communications, but would like to request some more detailed descriptions as commented below:

We thank the referee for appreciation of our work, and the insightful questions which helped to improve the clarity of the manuscript.

1. The colors in the background of figure 3a seem to suggest the ground state of the molecular cluster depending on tip position. If this is correct, I wonder why the background in section 3 with one electron on the cluster has three distinct areas (010, 010, 100) rather than the 010 and 001 being at the same energy? Or am I missing something? Similar questions apply to region 2 and 4. In any case, a more detailed explanation of the areas would be helpful.

   The background in Fig. 3a does not indicate ground state, but time averaged stationary occupancy of the many-body states resulting form solving the Pauli master equations (PME). These are out-of-equilibrium occupancies which do not necessarily correspond to the ground state. We acknowledge that this information may be a bit redundant as the same occupancy is visualized also by the thickness of the lines for each many-body energy, however we found it difficult for reader to follow these lines, especially when lines are overlapping, therefore we decided to plot it also on the background via "stacked plot". In this stacked plot the occupancies are normalized to sum up to one. Referee is right that states 010 and 001 are degenerate along this symmetric profile and therefore their occupancy should be identical, which is really the case in the figure (although it may be not immediately apparent on the first glance from the stacked plot visualization). We acknowledge that despite our effort the visualization is confusing without proper explanation, therefore we expanded the discussion and figure description.

2. The explanation of the contribution to the tunneling current in figure 3b is not entirely clear to me. Are the colored contributions relative contributions or some absolute values? The current seems to vary already along the red molecule. Why? Where does the exponential dependence enter or where can one see it in the plot?

The description of figures and their meaning is minimal in the paper. More details should be presented in the revised version.

In Fig. 3b we plot the absolute tunneling current (in arbitrary units). The variation in the areas far from charge rearrangement is dominated by value of tunneling matrix element which is modeled by an exponential of distance from each site (in simple model in Fig. 3) or by convolution of this exponential with orbital shape (in Fig. 2). To address specifically referee question about the dominance of the $011 \rightarrow 111$ *transition when tip is over red molecule: The current increases exponentially as the tip approaches the molecule, until it suddenly changes due crossing of many-body levels, resulting in charge rearrangement. We decided to plot these absolute rather than relative (normalized) contributions of different tunneling processes since the spatial variation of the current is important to explain shading within the rings (Fig. 3c) as well as intensity of the dI/dV spectra (Fig. 3d) and charging rings (Fig. 3e). We acknowledge that image description was insufficient and we expanded it accordingly.*

3. Very early on in the manuscript, the authors assume the ring to be a discharging process. They may want to stress that the molecules are charged in their ground state as can be deduced from the YSR spectra already at this point, possibly even including the spectra in the main manuscript. They may also want to comment on the origin of the splitting of the YSR states.

   We thank the referee for this valuable suggestion. We reorganized the main text to point out the connection between the radical nature of the molecules and the emergence of the YSR states from the beginning. We also mention the source of the splitting in the main text. Nevertheless, we prefer to present the complete analysis of the YSR spectra in the Supplemental Information to keep the focus of the main part on the high-energy behavior.

Referee 2

The authors study clusters of TBTAP molecules deposited on a superconducting substrate Pb(111) surface, and laterally manipulated using an STM tip. The molecules are arranged with atomic precision into an equilateral triangle configuration, with 1nm distance separating molecule centres. Low-temperature scanning tunnelling spectroscopy is used to create a real-space map of the tunnelling current, which provides insights into charging/discharging effects of the molecular cluster, due to the electric field of the STM tip and applied voltages. A complex structure of charging rings is uncovered, including chiral features and regions of negative differential conductance. In particular, charging rings are shown to arise from internal charge redistribution within the cluster without changes to the total charge.

The authors perform careful and sophisticated modelling of these effects, taking into account quantum many-body effects beyond the often-employed single-particle picture. They model the triangular molecular cluster as a three-impurity Anderson model, including both on-site Coulomb interaction U and capacitive inter-site repulsion W, as well as molecule-substrate hybridization and coupling to the STM tip, and modulations to the local electric field coming from the STM tip. This is an impressive level of theory –

absolutely essential to capture many-body effects and Coulomb blockade as well as experimentally observed transport features at low temperatures. In equilibrium, NRG is used to characterize the system, which fully takes into account interaction-renormalization effects and molecule-substrate entanglement. This treatment goes beyond weak coupling approximations and captures non-Markovian effects. However, it is restricted to equilibrium. To capture finite-bias transport and non-equilibrium occupation effects, the authors also employ the PME method, which is Markovian and valid only at weak coupling, but does capture essential features of the experiment in the appropriate limit. It should be noted that this is already state-of-the-art and no computational method exists to treat strong correlations, strong substrate coupling, and finite bias exactly and on an equal footing. Therefore I find this treatment impressive. The good match between theory and experiment also validate and justify the approach and methodologies used, which I believe are entirely appropriate.

The key findings are that many-body collective effects due to strong electron correlations, produce complex STS signatures and charging effects that cannot be described within a single-particle picture. This is an impressive work, with interesting results, and an excellent marriage of theory and experiment. The manuscript itself is also well-written. This work is entirely suited for publication in Nature Communications (or Nature Physics for that matter). I have the following questions and suggestions to improve the manuscript and analysis:

We thank the referee for the positive assessment of our work and for the constructive questions that helped to improve the quality of our manuscript and also helped us to put the paper into better perspective with regard the previous works.

1. In terms of the context of the present work, it would help to mention highly relevant related work on quantum dot trimers (triple quantum dots). Although the physical system and experimental measurement is different, the underlying theoretical model is basically the same. In particular, triangular TQDs are well-known to have multiple collective states (for example overall spin doublet states with different chirality but the same electron filling) which are induced by strong correlations. Importantly, the ground state can switch between these, and this shows up in transport. See e.g. PRB 80, 155330 (2009); PRB 79, 085124 (2009); PRB 81, 075126 (2010).

   We thank the referee for pointing out the relevant publications on triple QDs by Numata et al., and Mitchell et al. that we omitted. We added them to the list of references.

2. As the authors point out, transport in multi-orbital systems (molecular or QD) is often discussed in terms of simplistic single-particle quantum interference. However, for systems which show Coulomb blockade or Kondo due to strong electron correlations, this is totally insufficient to explain basic charging or transport effects. This is an important point, but it should be pointed out that quantum interference effects are also important for interacting systems – but one must now think about many-body quantum interference in Fock space. The proper transport problem is not simply about rearranging charges in many-body states, but about complex transition amplitudes between Dyson orbitals. The present findings fit in nicely with

these theories, but I suggest placing the current work in the proper context. See e.g. PRB 90, 125413 (2014); Nature Communications 8, 15210 (2017); PRL 133, 076501 (2024).

It is true that in interacting multi-orbital systems, transport can involve quantum interference between different many-body tunneling amplitudes. Our simulation results, however, are consistent with the scenario of vanishing direct hopping between orbitals on different molecules, in which all interactions are taking effect via the substrate or the capacitive coupling. The only process that can lead to interference effects is the indirect hopping $(i) \rightarrow (\mathbf{k}) \rightarrow (j \neq i)$ *via the substrate, leading to an effective exchange. But as our PME simulations are performed at high voltages* $V > 400\,meV$*, the injection of such 'hot' electrons into the system breaks the phase coherence in the system which could lead, e.g., to the Kondo effect. As a result, the dynamics at larger voltages can be interpreted in terms of transitions between charge configurations, with different transport channels. We expanded the the main text and the list of references to better relate our findings to previous works.*

3. Each TBTAP molecule is treated as just a single quantum orbital (Anderson impurity). Obviously a full ab initio treatment of the entire molecule (or cluster) would be far out of reach when taking into account interactions, but how/why is a single-orbital approximation appropriate? What about the real-space structure of the molecular orbital in question? The approximation appears to be justified post hoc by comparison with experiment, but what is the physical reason for this drastic simplification?

   Our choice of theoretical model was from the start motivated by simplicity, and the Anderson impurity model with single level on each site solved using PME was the simplest level of theory capable to explain all experimentally observed features. Attribution of relevant charge states to occupancy of SOMO orbital of the molecule is mainly in accordance to existing literature on similar systems and experimentally observed STM images consistent with nodal structure of respective orbital. The presence of other orbitals, that we consider fully occupied or empty, is hidden in the values of the model parameters, through renormalization of the energy level and screening of the interactions. Nevertheless, we agree with the referee, that real charge rearrangement may be more complex and involve multiple molecular orbitals (resp. be described by Dyson orbital as suggested be referee). However, the effective Hamiltonian model of current form involving on-site energies, inter-site Coulomb coupling and hopping to the tip and substrate, is still valid even if physical meaning of these effective molecular charge states is modified. To answer the second part of the question, the real-space structure of the SOMO orbital was calculated using DFT (Suppl. Fig. 5b) and incorporated into the results presented in Fig. 2, to simulate the chiral and nodal structure observed in the experiment. In comparison, the effects of the orbital symmetry were omitted in Suppl. Fig. 8.

4. Why is a superconducting substrate used, and what role does it play here? It would seem to me that this could complicate transport signatures significantly, relative to a standard metallic single-molecule transistor setup.

Our work is a continuation of previous studies on TBTAP molecular layers [Nano Lett. 25, 15206 (2025)] and smaller assemblies such as individual molecules, dimers, and short chains [ACS Nano 19, 3403 (2025)] on the same substrate, which enables a direct comparison across different assembly sizes and geometries. We chose Pb(111) because it provides a well-established and highly reproducible platform for assembling and manipulating TBTAP-based molecular structures with atomic precision.

In the earlier works, superconductivity and the associated YSR physics played a central role. In contrast, the focus of the present manuscript is on the non-trivial charging behavior and negative differential conductance of a different assembly motif. The key transport features discussed here occur on an energy scale and in a bias regime where they can be treated independently of the low-energy superconducting response of the substrate. In this sense, the superconducting nature of Pb(111) does not determine the mechanism proposed in this work, although it generates additional low-energy signatures at the gap edge (see Suppl Fig. 3).

For completeness, we include in the Supplemental Information an analysis of the YSR states in triangular trimers, which shows that these low-energy superconducting features are interesting in their own right but are not required to account for the main charging phenomena discussed in the manuscript. In addition, Pb(111) is particularly advantageous experimentally because it enables reliable molecular manipulation and stabilization of the assemblies, which is essential for constructing and probing the structures studied here.

5. For the NRG studies, more details of the implementation and calculations performed must be provided in the Methods or Supplementary Information section. All model and NRG parameters as well as further corroborating data should be provided.

   Most relevant parameters are provided in the Methods section, and we have expanded the Supplemental Information to include additional details on the NRG calculations. In particular, we now provide the full model definition as implemented in SNEG/NRG Ljubljana, along with a complete list of all parameters used in the NRG calculations. Furthermore, the scripts required to run the NRG code, as well as all relevant data, are available in the Zenodo repository.

   For the theory section in the Supplementary Information, what is the justification for the transport formulae used? Why not use Landauer-Buttiker or Meir-Wingreen for a more rigorous description?

   The Landauer-Büttiker (LB) approach is a single-particle, coherent transport framework to solve a scattering problem which assumes that the electrons are non-interacting and fails to capture the many-body correlations. Therefore, it is not applicable for a strongly correlated system with large on-site Coulomb repulsion as the one we are dealing with. Also, we used the LB approach in our first exploratory calculations and it completely failed to describe the NDC features, as expected. The Meir-Wingreen formula can be used to incorporate the many-body correlations, but it would require the knowledge of the interacting self-energy (or Green function) that would have to be calculated by a sophisticated numerical many-body solver (e.g., the NRG that was used to treat the YSR physics). Such calculations, especially for higher bias

voltages, are computationally demanding and do not allow for systematic parameter scans. On the other hand, the master equation approach is a many-body technique that calculates the evolution of the density matrix (or occupancy probabilities) and can natively handle strongly correlated systems in a simple, computationally efficient and fully tractable way and can explain all the experimental features. As an example, during the model parameter fitting, we plotted large number of two-dimensional maps of 10000 pixels (as in Fig. 2) each that were calculated using PME within seconds. Similar calculation would be unfeasible using NRG or similar techniques.

In the section detailing the model, more discussion should be provided on why this form of the hybridization matrix is used. Given the off-diagonal substrate hybridization terms, do we end up with a complicated multi-channel Kondo model? Or is it tacitly assumed that all three molecules couple to the same bath orbital, making it a single-channel problem (plus the STM tip). If the latter, what is the justification for this? Why are there no off-diagonal hybridization terms with the tip?

In our calculations we made the assumption that all three molecules are coupled to the same lead orbital, leading to an effective single-channel model. This allowed us to treat the YSR physics using NRG, which would be impossible for the three-channel problem due to computational complexity. As suggesting by the referee, this is a simplifying approximation. In particular, it does not capture spatially dependent correlations mediated by the substrate, which would require an explicit multichannel description. Such effects may become relevant, for example, in the detailed analysis of in-gap spectra. We are currently investigating this more general scenario. While multichannel effects may influence certain spectral features, they are not expected to significantly affect the charge rings that appear at the scale of hundreds of mV, that is the primary focus of the present work.

Concerning the coupling to the tip, we chose to ignore off-diagonal hybridizations with the tip as we consider the coupling to the tip to be much smaller than to the substrate and it scales exponentially with distance, making such terms negligible almost everywhere except special points like at the center of the cluster or directly between two sites, where the distance to two different sites is small and comparable. This is of course another approximation and the full model should contain also the off-diagonal coupling terms with the scanning tip.

6. For the PME calculations, how is the weak-coupling approximation justified and why does it work so well? In particular, the temperature is rather low, and so system-substrate entanglement and renormalization effects would be generically expected (these are the effects captured by NRG). The physics should be non-Markovian at low T, even for weak coupling. Indeed, we are told that $\Gamma > 10T$ and so this is really not the sequential tunnelling regime. How is Gamma quantified here and what is its physical meaning? One explanation could be that if by Gamma you are taking the standard quantum impurity definition $\Gamma = \pi\rho_0 V^2$ where V is the tunnel-coupling between system and bath and ρ_0 is the bath density of states at the Fermi level... then ρ_0 is strongly suppressed by the superconducting gap of the substrate and tip.

Thus one suppresses Kondo and other strong-coupling effects and weak-coupling methods are remarkably accurate, see e.g. PRL 107, 256802 (2011).

We thank the referee for this important question. The size and shape of the charging rings is given by the electrostatic field of the STM tip and the molecule rather than the coupling strength, which mostly tunes the finer features of the charging pattern. Therefore, the results of the rings fitting do not depend much on the value of Γ*. The definition of* Γ *is indeed given by* $\Gamma = \pi\rho_0 V^2$*, as stated in the Suppl. Information below Eq. (5), but we note that the superconducting nature of the surface is not relevant here, as the gap is very small (*≈ 1.3 *meV) compared to the typical tip voltages (*$0.1 - 1$ *eV).*

The value of $\Gamma_s = 10 - 20$ *meV is a low-energy parameter fitted at equilibrium at the energy scales relevant for YSR spectroscopy, whereas the coupling in PME is an effective rate parameter governing nonequilibrium transport at voltages of order hundreds of mV. One can therefore expect that they are distinct. Based on the recent study of the electron–vibration coupling (EVC) of TBTAP molecules on Ag(111) surface [Nat. Commun.* ***14****, 5956 (2023)] we suspect that EVC can significantly contribute to the good agreement of the weak-coupling approximation with experiment. The reason is that EVC can reduce the effective coupling through Franck-Condon dressing. It was shown in the mentioned paper that, assuming just a single vibronic mode of the molecule, a dimensionless EVC strength is* $\lambda/\Omega_0 = 2.4$*, which is significant. Although generalization to high voltage regime is not straightforward, one can expect a strong reduction of the effective molecule-lead coupling, possibly by several orders of magnitude, just from this effect. We emphasize, however, that this mechanism is invoked here only as a plausible explanation of the suppression of effective* Γ *in the PME description because vibrations are not treated explicitly in our model and go beyond the scope os this paper. We have now added an explanation at this point in both the main text and the Supplemental Information.*

7. It is stated that the "full" Hilbert space of the molecular cluster is $4^3 = 64$ states but that the large Coulomb interaction suppresses double occupancy and reduces this to $2^3 = 8$ which is tractable. I would make a few comments here. First, it should be made clear that the actual Fock space dimension of each molecule is enormous and far beyond reach of exact diagonalization or ab initio methods; the local Fock space dimension of 4 is already a vast simplification by keeping just the SOMO (and this will be an approximation since there will be interaction effect between all Mos). Secondly, $4^3 = 64$ is still a tiny Fock space in the context of many-body problems, and is fully tractable. The issue is, rather, that coupling to the substrate and STM tip beyond weak-coupling (Markovian) approximation leads to genuine non-perturbative many-body effects because now one has to deal with the lead electrons as well. This is exactly the physics taken into account exactly by NRG. Finally, I do not really understand the reduction to $2^3 = 8$ states. Precluding double occupancy leaves 3 states on each SOMO so I would have thought this is $3^3 = 27$ states remaining that need to be described (up, down, empty).

We performed calculations of the charging features with both finite value of $U =$

200 meV (inferred from older experiments), in which we consider 64 states, and a simplified calculation with infinite U, in which the doubly occupied states are prohibited and the spin degree of freedom is constrained. The differences in the results were negligible (Suppl. Fig. 7). Therefore, we used the simplified picture despite the fact that the full problem would be still tractable, because of both the computational efficiency of this approach and the simpler reasoning needed to describe the experimental results.

As we are not dealing with any effects of magnetic field, spin-orbit coupling, or the Kondo physics (due to high voltages) the spin degree of freedom is not relevant and the model of spinless fermions interacting through the inter-site coupling W with only $2^3 = 8$ states is adequate. The non-perturbative effects mentioned by the referee are indeed important for the low-energy (YSR or Kondo) calculations, which were performed using NRG at finite U.

8. The mechanism for the tip-induced charge reorganization on the trimer is interesting and obviously a crucial for the story. In the $N = 2e$ sector, the interpretation presented by the authors is that the tip potential pushes out the electron on the site below it, onto the remaining two molecules. However, if there is no direct tunnelling and we have effectively 3 degenerate, localized 2-electron states $|1, 1, 0>$, $|1, 0, 1>$, $|0, 1, 1>$ then this charge reorganization (which I am happy to accept does happen, given the experimental evidence!) must happen by tunnelling into and then out of the substrate. Is that so?

   We agree with the referee's interpretation that, in the absence of direct inter-molecular hopping, charge redistribution within the trimer must proceed via the substrate. In our modeling and parameter regime, the direct tunneling between molecules is indeed negligible, so the reorganization is mediated by tunneling processes involving the substrate.

   My issue here is that the substrate is superconducting and therefore at low T can only accept or release cooper pairs. Single-electron tunnelling is suppressed inside the SC gap Delta. So the charge reconfiguration is maybe happening by tunnelling a **pair** into the substrate, and then back out into different molecule states? Another possibility is that the actual quantum $N = 2e$ state is a superposition of $|1, 1, 0>$, $|1, 0, 1>$, $|0, 1, 1>$ from the start, and so the tip field is just re-adjusting the relative weights of these rather than causing explicit tunnelling events. The situation is even more confusing for $N = 1e$, because here there is no pair that can tunnel directly. Here is seems more likely that the $N = 1e$ state is actually a quantum superposition of "classical" states with 1e on either one of the molecules. This should be addressed and discussed in the manuscript and supporting evidence for the mechanism provided where possible.

   Regarding the role of superconductivity, we emphasize that the charge reorganization discussed in the manuscript is associated with the discharging rings observed at sample voltages exceeding 400 mV, which is far above the superconducting gap ($\Delta \approx 1.3$ meV). In this regime, single-electron tunneling into the continuum states of the substrate is allowed, and superconductivity does not impose a significant con-

straint. Therefore, the charge redistribution can proceed via standard single-electron tunneling processes. We have now stressed this in the main text as well as in the relevant section in the Supplemental Information.

However, the situation is qualitatively different within the superconducting gap, where single-electron tunneling is suppressed and pair processes or coherent superpositions become relevant. In this subgap regime, the many-body states are indeed more appropriately described as quantum superpositions of different particle configurations (for example atomic or molecular BCS singlet states). This is particularly important for the interpretation of YSR states, where multiple nearly degenerate singlet and doublet configurations can arise due to the proximity effect.

We partially address these subgap processes in the Supplemental Information. However, a complete treatment of the interplay between multi-configurational states, superconductivity, and YSR physics in this system is beyond the scope of the present work and will be the subject of future study.

Referee 3

In this manuscript, Li et al report a work of using STM tip to spatially map out characteristic peaks and dips in differential conductance (dI/dV) on trimer clusters of TBTAP molecules on a Pb(111) substrate. Using highly complicated theoretical modelling, they provide an explanation of the experimental observation. They argue, instead of the common mechanism of charging and discharging, the dI/dV peak/dip are attributed to charge rearrangement among the three molecules in the cluster. They conclude that this work "shed new light on fundamental quantum many-body effects in strongly interacting molecular systems and open pathways for engineering novel electronic phases by controlling cluster topology and the electrostatic environment." The experimental data presented in the manuscript are of very high quality. However, the theoretical arguments are very complex and difficult to follow for the reviewer. The following comments are mostly about the theoretical arguments:

1. On Page 2: the reviewer finds "we directly visualize discharging processes and characteristic Coulomb ring patterns associated with single-electron charging events, including the onset of robust negative differential conductance (NDC) near the center of the trimer." And "the (dis)charging rings and conductance discontinuities do not correspond to a change in the total charge of the trimer. Instead, they originate from subtle intra-cluster charge rearrangements, where electrons are redistributed among the molecules without altering the net charge." So, it seems that the trimer does not gain or loss charge in the whole process. If this is the case, the author shall not use charging/discharging, which confuses the readers.

   Referee is right that meaning of word "charging" is nuanced in this situation and need to be carefully discussed. While we stress that so-called "charging rings" does ***not always*** *correspond to change of total charge of the cluster (as indicated by sharp dI/dV peaks between the areas of same total charge visible e.g. in Fig. 1e,f and Fig. 3d), they still correspond to change of charge state of individual molecules*

in cluster (meaning total charge is conserved when one molecule gain charge while other molecule loose charge). It is worth noting that some charging rings (i.e. dI/dV peaks) still correspond to change of total cluster charge, as is visible on the same figures. We clarify this better in the manustript.

2. On Page 4, the reviewer reads "charging the cluster with two electrons to −2e more energetically favorable than charging it with three electrons to −3e. and "Upon applying a sufficiently high bias voltage (sample voltage $V_s = 0.6$ V when tip is at the center of the cluster), an additional electron is repelled from the cluster, leading to a net charge of −1e, meaning that only one site remains occupied." This is the key point. Experimentally, these charge states can be confirmed using Kelvin parabolas showing the LCPD using nc-AFM. To unambiguously verify the proposed mechanism, such measurements are essential.

   *We thank the reviewer for this valuable suggestion. We agree with the referee that $\Delta f(V)$ measurements would provide an additional information regarding the charge-state control in the triangular molecular assembly. Such measurements can corroborate the charge-state control from anionic to neutral TBTAP by capacitive coupling with the tip field, which signature is a dip in the $\Delta f(V)$ spectrum. Note that we have already observed such signature for anionic TBTAP in their self-assembly in a previous publication (C. Drechsel et al. Nanoscale Horiz., **10**, 2365, 2025), which is identical to the peak observed in dI/dV spectra.*

   Regarding local contact potential difference (LCPD) measurements obtained by tracking the top of the $\Delta f(V)$ parabola, it would also provide an additional identification between charged and neutral molecules within the molecular clusters. Similarly, observing both YSR states in the superconducting gap and their discharge by tunneling spectroscopy yields an unambiguous assignment of their charge state in the ground state.

   Finally, we would like to emphasize that the present work focuses not only the identification of a static charge state, but also the emergence of negative differential conductance (NDC) from a bias-driven nonequilibrium charge rearrangement within the coupled molecular cluster. Therefore, LCPD measurements would mainly serve as complementary support for the charge-state assignment within the molecular trimers, rather than as a complete standalone verification of the transport mechanism only obtained through tunneling measurements.

   In the revised manuscript, we have clarified this point and now explicitly state that the proposed charge configurations are inferred from the combined evidence of the spatially resolved bias-dependent dI/dV measurements, including the evolution of the discharging rings, together with the quantitative many-body transport calculations, which consistently reproduce the observed NDC behavior.

3. The total charge of the cluster for different regions is marked in Figure 3d, showing at the two sides, the charge is 2. However, Figure 3b shows at the left side the charge is 2 as labelled with "011", while at the right side the charge is 1 as labelled with "001". The labels are confusing. Also, Fig. 3b shows $011- > 111$, but this is not discussed in the text.

Referee probably refers to blue parabola on the left side of Fig. 3d which has really charge $n = 2$ on both sides. We expanded the range of tip positions in Figs. 3a-d to visualize this transition. The tunneling process between where electron from the tip occupy the red molecule thus transforming state 011 to 111 is really dominant component of tunneling current on the left most region of our 1D and 2D scans. In the previous version we omitted the discussion of this process as it was not essential for explaining negative differential resistance which was our main point. We extended the discussion in the manuscript to mention this process.

4. The discussion of Fig. 3a is very brief and general, only five lines on page 4. Based on these five lines, the reviewer does not understand the very complex features of Fig. 3a. Similar situation is encountered for Fig. 3b.

   We thank the referee for pointing out this issue. We acknowledge that image description was insufficient and therefore we expanded the relevant part of the main text.

5. It is not clear for the reviewer how the bias voltage of the STM junction is treated in the modelling. Considering the major arguments heavily rely on the modelling, a concise presentation of the Hamiltonian and/or schematic configuration of the system shall be presented in the main text.

   The voltage of the junction is treated as a shift of the local energy levels, $\varepsilon_i(\mathbf{r}) = \varepsilon_{id} + \nu_i(\mathbf{r})$ *where* $\nu_i(\mathbf{r})$ *is the energy shift due to the presence of the electrostatic field of the scanning tip at position* $\mathbf{r}$*. We have left this discussion for the Supplementary Information as by our opinion including the rather complicated Hamiltonian and all the related discussion into the main text would dilute the message of the paper and distract the reader from the main points. We updated the manuscript to point reader who is interested in the details of the modeling procedure to the right section of the Supplementary Information. We also added a schematic plot of the setup for clarity.*

List of changes

- Extended Data Figures were moved to Supplementary Information (SI) to accommodate the Nat. Commun. guidelines (the manuscript was transferred from Nat. Phys.).
- Added Refs. [15]-[21], [41], [44-47]
- Reorganized the main part to better point out the connection between the emergence of the YSR states and the charge state of the molecular cluster.
- Extended Figs. 3a,b to a larger interval of tip positions to provide a more complete understanding.
- Extended discussion of Fig. 3.

- Extended the discussion about quantum interference effects and the possible source of coupling reduction.
- Added and explanation for the reduction of a the spinful model to a model to interacting spinless fermions.
- Added a sentence on the effect of the bias voltage on the model.
- Added Suppl. Fig. 1 with the schematics of the setup.
- Added more details about the NRG implementation SI.
- Added few sentences discussing bias-dependent frequency-shift and local contact potential difference measurements as complementary evidence.
